# Design, Synthesis, and Acaricidal Activity of Phenyl Methoxyacrylates Containing 2-Alkenylthiopyrimidine

**DOI:** 10.3390/molecules25153379

**Published:** 2020-07-25

**Authors:** Shulin Hao, Zengfei Cai, Yangyang Cao, Xiaohua Du

**Affiliations:** Catalytic Hydrogenation Research Center, Zhejiang Key Laboratory of Green Pesticides and Cleaner Production Technology, Zhejiang Green Pesticide Collaborative Innovation Center, Zhejiang University of Technology, Hangzhou 310014, China; caizengfei@zjut.edu.cn (Z.C.); yycao@zjut.edu.cn (Y.C.)

**Keywords:** phenyl methoxyacrylates, thioether, synthesis, acaricidal activity, structure activity relationships

## Abstract

A series of novel phenyl methoxyacrylate derivatives containing a 2-alkenylthiopyrimidine substructure were designed, synthesized, and evaluated in terms of acaricidal activity. The structures of the title compounds were identified by ^1^H NMR, ^13^C NMR and high-resolution mass spectra (HRMS). Compound (*E*)-methyl 2-(2-((2-(3,3-dichloroallylthio)-6-(trifluoromethyl)pyrimidin-4-yloxy)methyl)phenyl)-3-methoxyacr-ylate (**4j**) exhibited significant acaricidal activity against *Tetranychus cinnabarinus* (*T. cinnabarinus*) in greenhouse tests possessing nearly twice the larvicidal and ovicidal activity compared to fluacrypyrim. Furthermore, the results of the field trials demonstrated that compound **4j** could effectively control *Panonychuscitri* with long-lasting persistence and rapid action. The toxicology data in terms of LD_50_ value confirmed that compound **4j** has a relatively low acute toxicity to mammals, birds, and honeybees.

## 1. Introduction

Around 300,000 to 500,000 species of mites are distributed all over the world. Phytophagous mites are recognized as one of the most difficult pest communities to control, which can harm more than 150 crops, especially cotton, citrus, apples, vegetables, tea and flowers, causing great losses to agriculture and forestry every year [1,2,3,4,5,6,7,8]. On the other hand, due to the improper and frequent use of pesticides and a series of characteristics of mites themselves, such as the short generation cycle, rapid reproduction, high inbreeding rate, and high mutation rate, mites have already developed more and more serious resistance to the existing acaricides [9,10,11]. It is reported that 53 species of resistant ticks exist in the world [6]. Therefore, there is an urgent need for continually discovering and developing new acaricides with improved activity to safeguard crops against mites.

Compounds containing the methoxyacrylate moiety are widely applied as agrochemical acaricides [12]. Fluacrypyrim, the first strobilurin acaricide, was discovered by (Badische Anilin-und-Soda-Fabrik) BASF and developed by Nippon Soda [13]. It can effectively control all the growth stages of spider mites with a mode of action inhibiting mitochondrial electron transport in complex III within the respiratory chain [14]. Besides, Pyriminostrobin invented by the Liu group at Shenyang Sinochem Agrochemicals R&D Co., Ltd. and HNPC-A3066 discovered by Liu et al. at Hunan Research Institute of Chemical Industry, are another two typical strobilurin acaricides [15,16].

**Figure 1:**
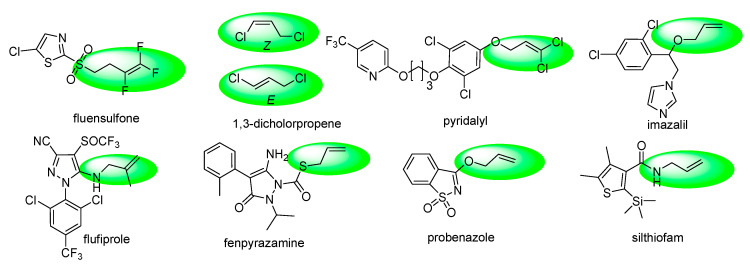
The existing pesticides containing alkenyl/haloalkenyl moieties.

In recent years, molecules containing alkenyl and haloalkenyl moieties have played an important role in the R&D of new agrochemical chemistry. It is noteworthy that alkenyl/haloalkenyl is a multi-functional group that has been incorporated into essential structural motifs to improve the physical, chemical, and biological properties of many biologically active molecules, such as agricultural nematocides fluensulfone, 1,3-dicholorpropene; insecticides pyridalyl, flufiprole; fungicides fenpyrazamine, silthiofam and imazalil, and bactericides/fungicides/plant activators probenazole (Figure 1) [17]. Furthermore, the synthesis of new pyrimidine derivatives bearing the alkenyl/haloalkenyl group has attracted much attention from many organic chemists. Substituted 2- (alkenyl/haloalkenyl)thio)-pyrimidine showed good herbicidal, fungicidal and insecticidal activity [18,19,20,21,22,23].

**Figure 2:**
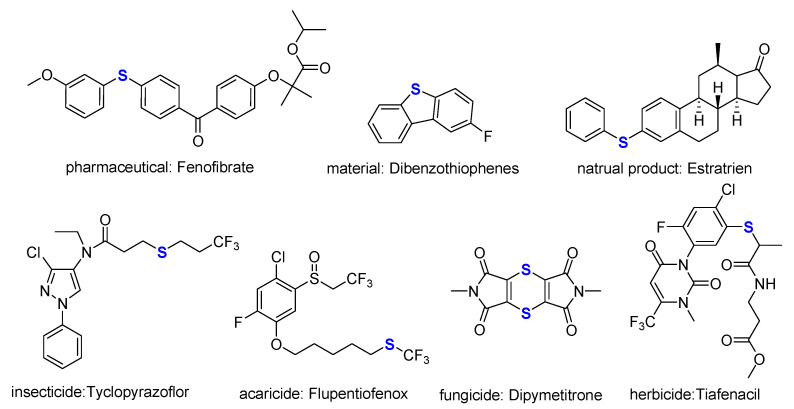
Structures of the reported sulfur-containing compounds.

Organosulfur compounds are of fundamental and immense importance in organic chemistry. Sulfur-containing frameworks containing sulfur-containing heterocycles, sulfonamides, sulfonylureas, thioethers, sulfones, thioesters and thiocarbonyl compounds, have attracted considerable interest due to their unique physical and chemical properties [24,25,26,27], as well as their physiological activities [28,29,30]. Remarkably, in recent decades, thioether has emerged as the key functional groups widely used in natural products [31,32,33], medicine [24,25,34], pesticides [35,36], bioinformatics [37] and materials [28,38,39] (Figure 2). As a result, thioethers-containing molecules are attracting more and more attention based on their variety of biological activities [29].

**Figure 3:**
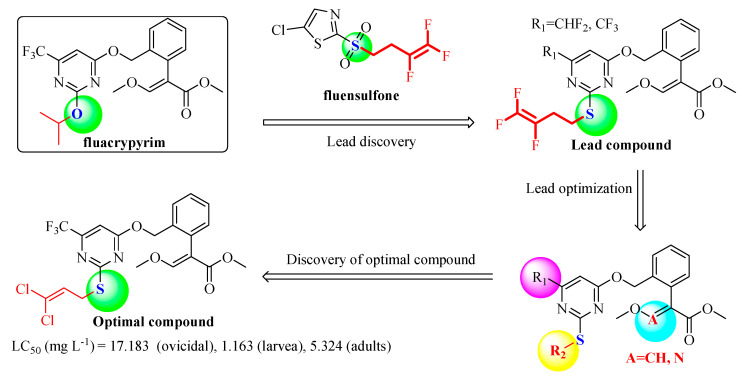
Design strategy of the target compounds.

**Figure 4:**
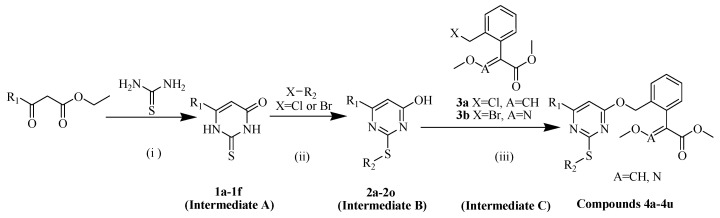
Synthetic procedure for the title compounds. Reagents and conditions: (**i**) EtONa, EtOH, reflux, 4–6 h. (**ii**) K_2_CO_3_, *N*,*N*-Dimethylformamide (DMF), 40–50 °C, 3–5 h. (**iii**) K_2_CO_3_, DMF, 60–80 °C, 3–5 h.

Traditional synthetic procedures of fluacrypyrim involve a long synthesis step, low yield and poor cost performance. With an aim to find novel strobilurin acaricides with improved acaricidal activity and cost performance, and follow the isosteric design, we try to introduce the thioether group into strobilurin fluacrypyrim and construct a pyrimidine substructure bearing alkenyl/haloalkenyl at the same time. A series of novel strobilurin compounds were designed and synthesized (Figure 3). The synthetic routes for the target compounds **4a**–**4u** and for the intermediate **2a**–**2o**, are displayed in Figure 4, and have the advantages of convenient synthesis, simple post-processing, and high yield. The detailed synthesis, structure and activity relationship (SAR), and acute toxicology data are presented, which would provide the basis for further research.

## 2. Results and Discussion

### 2.1. Chemistry

The target compounds were characterized by ^1^H NMR, ^13^C NMR, melting point, high-resolution mass spectra (HRMS) analyses and some compounds were characterized by ^19^F NMR analyses. All the spectral and analytical data were consistent with the assigned structures (Supplementary Materials).

It is well known that the homologous elements in the same group with a higher atomic number have a lower electronegativity, so they have the stronger electron-donating ability and thus greater nucleophilicity [40,41]. Therefore, under the same conditions, the reaction between the thiol at the 2-position of the pyrimidine and halogenated alkenes could be well controlled by setting the reaction temperature between 40 and 50 °C, while the reaction between the halogenated alkenes and the hydroxyl at the 4-position of the pyrimidine could be reduced. The intermediate B is the key to synthesize the target compounds, and their synthesis procedures are shown in Figure 4. The physical properties of intermediates (**1a**–**1f** and **2a**–**2o**) and target compounds (**4a**–**4u**) are presented in Table 1 and Table 2.

### 2.2. Acaricidal Activity

The acaricidal activity results of all the title compounds to control adult *Tetranychus cinnabarinus* (*T. cinnabarinus*) by using the spraying method in the greenhouse are listed in Table 3.

**Figure 5:**
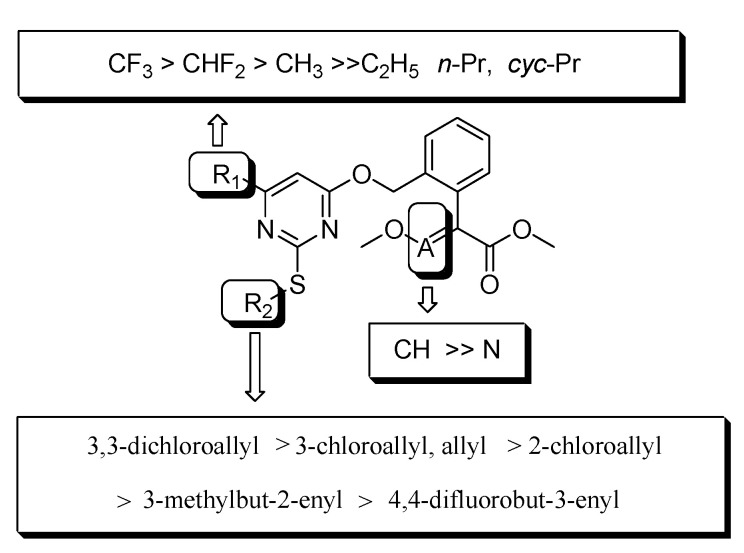
Structure activity relationships for the compounds **4a**–**4u** against adult *T. cinnabarinus.*

In order to explore the bioactivity of our design thinking, considering the importance of alkenyl and fluorine [42,43], we firstly import the moiety 4,4-difluorobut-3-enyl of nematocide fluensulfone to acaricide fluacrypyrim while keeping the R_1_ group of an electron-withdrawing and hydrophobic CF_3_ and CHF_2_, and *β*-keto esters were used as the starting material to obtain the compounds **4a** and **4b** (Table 3). The bioassay results indicated that the compound **4a** (R_1_ = CHF_2_) displayed around 90% control against adult *T. cinnabarinus* at 500 mg L^−1^, which was almost equal to the commercial fluacrypyrim showing 100% control at the same concentration. However, compound **4b** (R_1_ = CF_3_) did not exhibit any activity even at 500 mg L^−1^. Based on the above result, the compound **4a** was regarded as a primary lead compound for the further optimization focusing on the R_2_ group targeting *T. cinnabarinus*, while fixed R_1_ as CHF_2_ plus CF_3_ considered fluacrypyrim-bearing CF_3_ itself, although **4b** had no activity. The modification on R_2_ was paid attention to for the allyl and the series of chloroallyl instead of 4,4-difluorobut-3-enyl, such as the 2-chloroallyl, 3-chloroallyl, 3,3-dichloroallyl, Yes 3-methylbut-2-enyl. Thus, the compounds **4c**–**4k** were synthesized to evaluate the effect of the different R_2_ substituent on acaricidal activity (Table 3). Surprisingly, these compounds exhibited obviously better acaricidal activity than the lead compound **4a**; among them, the acaricidal activity of the compounds **4c**, **4f**, **4i** and **4j** against adult *T. cinnabarinus* was comparable to fluacrypyrim at a lower concentration of 20 mg L^−1^ reaching the control of 80 ± 4%. Especially, compound **4i** with a control effect of 70 ± 7%, and compound **4j** with a control effect of 80 ± 4%, demonstrated a significantly higher acaricidal activity against adult *T. cinnabarinus* than fluacrypyrim with a control effect of 40 ± 7% at the same concentration of 4 mg L^−1^. In general, when R_1_ was fixed as CHF_2_, the acaricidal activity of the target compounds against *T. cinnabarinus* increased according to the order of R_2_: allyl ≈ 3,3-dichloroallyl > 2-chloroallyl > 3-chloroallyl. For example, at a concentration of 20 mg L^−^^1^, the acaricidal activity of the compounds **4c** (R_2_ = allyl), **4d** (R_2_ = 2-chloroallyl), **4e** (R_2_ = 3-chloroallyl), and **4f** (R_2_ = 3,3-dichloroallyl) was 80 ± 2, 50 ± 3, 20 ± 7, and 80 ± 5%, respectively. Similarly, when R_1_ was fixed as CF_3_, the acaricidal activity of the target compounds against *T. cinnabarinus* increased according to the order of R_2_: 3,3-dichloroallyl > 3-chloroallyl > 3-methylbut-2-enyl > 2-chloroallyl > allyl (Figure 5). For example, at a concentration of 20 mg L^−^^1^, the acaricidal activity of the compounds **4g** (R_2_ = allyl), **4h** (R_2_ = 2-chloroallyl), **4i** (R_2_ = 3-chloroallyl), **4j** (R_2_ = 3,3-dichloroallyl), and **4k** (R_2_ = 3-methylbut-2-enyl) was 0, 50 ± 6, 80 ± 10, 80 ± 2, and 80 ± 3%, respectively. Additionally, the acaricidal activities of **4i** and **4j** were stronger than those of **4e** and **4f**, respectively, it seems that CF_3_ (R_1_) was required for the development of optimal acaricidal activity, rather than CHF_2_ (R_1_). Based on the analyses mentioned above, valuable information about the R_2_ group was revealed: that 3,3-dichloroallyl, 3-chloroallyl and allyl could be considered as optimal moieties for further optimization.

Encouraged by the above findings, R_1_ was still kept as CHF_2_, CF_3_ and R_2_ selected the three optimal moieties allyl, 3-chloroallyl and 3,3-dichloroallyl, respectively. We then turned our attention to the replacement of methoxyacrylate with methoxyiminoacetate. Six compounds (**4l**–**4q**) were prepared. To our disappointment, their acaricidal activity of the compounds **4l**–**4q** did not show better activity than their corresponding methoxyacrylate compounds, except the pairs of **4g** and **4o** (**4o** with a little higher activity than **4g**). Therefore, the methoxyacrylate group was maintained continuously in the following optimization.

Finally, based on the valuable SARs information achieved above, the R_2_ of 3,3-dichloroallyl and methoxyacrylate, we went back to modify the R_1_ of the pyrimidine ring with opposite electron effect groups like electron-donating methyl, ethyl, *n*-Pr and *cyc*-Pr. Unfortunately, all of the four compounds **4r**–**4u** did not display any activity, even at 500 mg L^−1^.

Therefore, the compounds **4i** (R_1_ = CF_3_, R_2_ = 3-chloroallyl, A = CH) and **4j** (R_1_ = CF_3_, R_2_ = 3,3-dichloroallyl, A = CH) with methoxyacrylate were regarded as the optimal structures with acaricidal activity at **4** mg L^−1^, respectively, which is significantly higher than that of commercialized control fluacrypyrim.

In order to further evaluate the potential of compounds **4i** and **4j**, their acaricidal activity comparison was conducted with fluacrypyrim as a control against all stages of *T. cinnabarinus* including adults, larvae, and eggs. As the data shown in Table 4, the acaricidal activity of compound **4j** against adult *T. cinnabarinus* (LC_50_: 5.324 mg L^−1^) was roughly equivalent to that of fluacrypyrim, (LC_50_: 4.178 mg L^−1^) and greatly higher than that of the compound **4i** (LC_50_: 138.626 mg L^−1^). More importantly, compound **4j** presented excellent ovicidal (LC_50_: 17.183 mg L^−1^) and larvicidal (LC_50_: 1.163 mg L^−1^) activity, nearly twice as much as fluacrypyrim (LC_50_: ovicidal, 43.332 mg L^−1^; larvicidal, 2.009 mg L^−1^). To date, compound **4j** has shown the best acaricidal activity, so we then examined the field-trial potential of a new acaricide candidate, compound **4j** ((*E*)-methyl 2-(2-((2-(3,3-dichloroallylthio)-6-(trifluoromethyl)pyrimidin-4-yloxy)methyl)phenyl)-3-methoxyacry-late).

### 2.3. Field Trials

In this study, the compound **4j** was selected as the most potent compound with the highest acaricidal activity against *T. cinnabarinus*. Field trials were carried out to confirm its field performance in November, 2018 in Nanning, Guangxi Province, China. The field trials (Table 5) indicated that the acaricidal activity of compound **4j** at 100 mg L^−1^ against citrus red mites was roughly equivalent to that of cyenopyrafen and slightly higher than that of cyetpyrafen, a newly commercialized acaricide from Sinochem Group. At a concentration of 100 mg L^−1^, the effect of compound **4j** on controlling the citrus red mite was 96.61, 97.36, 96.25, and 81.02% after 3, 7, 10, and 20 days of treatment, respectively, while cyenopyrafen revealed a controlling effect of 92.26, 97.85, 96.43, and 80.87% against *Panonychuscitri* after 3, 7, 10, and 20 days of treatment, respectively. At the same concentration, the effect of cyetpyrafenon controlling citrus red mite was also 97.70, 100, 99.38, and 89.79% after 3, 7, 10 and 20 days of treatment, respectively. The comparison of the above-treatment results indicated that the compound **4j** was a highly active and rapid-acting acaricidal compound, which can effectively control *Panonychuscitri* at an application rate of 100 mg L^−1^.

### 2.4. The Toxicity of Compound ***4j***

The toxicity of compound **4j** on primary mammals, birds, and honeybees was evaluated by The Safety Evaluation Center of Shenyang Research Institute of Chemical Industry (National Shenyang New Drug Safety Evaluation and Research Center, Shenyang, China) and Zhejiang Academy of Agricultural Science in 2019 as presented in Table 6. It was found that (1) the acute oral median lethal dose (LD_50_) of compound **4j** for rats is higher than 500 mg kg^−1^; (2) it has a low toxicity for birds (>2090 mg a.i./kg) and honeybees (>116 µg a.i./honeybee); and (3) it moderately irritates rabbits’ eyes.

## 3. Materials and Methods

### 3.1. Reagents and Instruments

The reagents were all high purity analytical or chemical grades, purchased from commercial sources and were used as received. All anhydrous solvents were dried and distilled by standard techniques before use. The melting points were determined by using an RY-1 melting point apparatus (TaiKe, Beijing, China). The ^1^H NMR, ^13^C NMR, and ^19^F NMR spectra were recorded utilizing an AV 400/500 spectrometer (Bruker, Karlsruhe, Germany) in CDCl_3_ or DMSO-*d_6_* solution using tetramethylsilane as an internal standard, and the chemical shifts (δ) were given in parts per million (ppm). High-resolution mass spectra (HRMS) data were obtained by employing Fourier transform ion cyclotron resonance mass spectrometry (FTICR-MS) (ionspec, 7.0 T). The reaction progress was monitored by thin-layer chromatography (TLC) on silica gel GF_254_ (Tianzhe, Qingdao, China), and the spots were visualized with ultraviolet (UV) light (Gonyi, Zhengzhou, China).

### 3.2. Synthetic Chemistry

All the title compounds were synthesized as described in Figure 4 (Page 6). General procedures for the preparation of the title compounds (**4a**–**4u**) and intermediates (**1a**–**1f** and **2a**–**2o**) according to the related literature [44,45,46] and spectra data of the title compounds (**4a**–**4u**) are provided in the Supplementary Materials. Intermediate **3a** was obtained from 1*H*-isochromene-3(4*H*)-one as the starting material via a multi-step synthesis referring to the literatures [47,48]. Intermediate **3b** was synthesized using a 2-methylbenzoic acid as the starting material [49,50].

#### 3.2.1. Synthesis of 6-Substituent-2-thioxo-2,3-dihydropyrimidin-4(1*H*)-one (**1a**–**1f**)

All of the thiouracil derivatives (R_1_ = CH_3_, C_2_H_5_, *n*-Pr, *cyc*-Pr, CHF_2_, CF_3_) were prepared as followed. To a 1000 mL three-neck flask equipped with a magnetic stirrer, a thermometer, and a dropping funnel, 500 mL of methanol and 59.4 g (1.1 mol) of solid sodium methoxide were added, and the mixture was then stirred at room temperature. After 0.5 h, 0.5 mol (93.0 g) of ethyl 4,4,4-trifluoro-3-oxobutanoate and 0.6 mol of thiourea were added to the flask, and the mixture was heated to reflux for about 4 h. Finally, a 2 M HCl aqueous solution was added to the flask until the reaction mixture was neutralized. The resultant solid was separated by filtration, then washed with water, and finally dried in a desiccator to produce 88.3 g of intermediate **1b** as a white solid at a yield of 90%; melting point: 247.5–248.2 °C; ^1^H NMR (500 MHz, DMSO-*d_6_*) δ 13.37 (br, 1H), 12.78 (s, 1H), 6.34 (s, 1H).

#### 3.2.2. Synthesis of 2-(3,4,4-Trifluorobut-3-enylthio)-6-(trifluoromethyl)pyrimidin-4-ol (**2f**)

Potassium carbonate (15.5 g, 98.0%, 0.11 mol) was added into a solution of 14.5 g (0.10 mol) of 6-trifluoromethyl-2-thiouracil (**1b**, R_1_ = CF_3_) in 100 mL of DMF, and the reaction mixture was stirred at 40 °C for 0.5 h, followed by the addition of 19.1 g (0.10 mol) of 4-bromo-1,1,2-trifluorobut-1-ene; then, the reaction mixture was heated to 50 °C for about 3 h and monitored by TLC until the reaction was complete; afterward, the mixture was poured into water, and a 2 M HCl aqueous solution was added to the flask until the reaction mixture was neutralized. The resultant solid was separated by filtration, then washed with water, and finally dried in a desiccator to obtain 28.0 g of the intermediate **2f** as a white solid at a yield of 92%; melting point: 82.6–83.7 °C; ^1^H NMR (500 MHz, DMSO-*d_6_*) *δ*13.33 (br, 1H, OH), 6.55 (s,1H, pyrimidy1-H), 3.26 (t, *J* = 9.0 Hz, 2H, CH_2_), 2.69–2.75 (m, 2H, CH_2_).

#### 3.2.3. Synthesis of 2-(2-Chloroallylthio)-6-(trifluoromethyl)pyrimidin-4-ol (**2h**)

Potassium carbonate (15.5 g, 98.0%, 0.11 mol) was added into a solution of 14.5 g (0.10 mol) of 6-trifluoromethyl-2-thiouracil (**1b**) in 100 mL of DMF, and the reaction mixture was stirred at 40 °C for 0.5 h, followed by the addition of 11.0 g (0.10 mol) of 2,3-dichloroprop-1-ene; afterward, the reaction mixture was heated to 50 °C for about 3 h and monitored by thin-layer chromatography until the reaction was complete; the mixture was then poured into water, and a 2 M HCl aqueous solution was added to the flask until the reaction mixture was neutralized. The resultant solid was separated by filtration, then washed with water (60 mL), and finally dried in a desiccator to form 25.0 g of intermediate **2h** as a white solid at a yield of 93%; melting point: 116.2–116.8 °C; ^1^H NMR (500 MHz, DMSO-*d_6_*) δ 13.36 (br, 1H, OH), 6.58 (s, 1H, pyrimidy1-H), 5.54 (s, 1H, CH_2_), 5.32 (s, 1H, CH_2_), 4.10 (s, 2H, CH_2_).

#### 3.2.4. Synthesis of (*E*)-Methyl 3-methoxy-2-(2-((2-(3,4,4-trifluorobut-3-enylthio)-6-(trifluoromethyl)pyrimidin-4-yloxy)methyl)phenyl)acrylate Compound **4b**

Potassium carbonate (1.55 g, 98.0%, 0.011 mol) and 2-(3,4,4-trifluorobut-3-enylthio)-6-(trifluoromethyl)pyrimidin-4-ol (**2f**) (2.5 g, 0.01 mol) were added to 50 mL of DMF and reacted for 1 h at 60 °C; then, 2.55 g (0.011 mol) of (*E*)-methyl 2-(2-(chloromethyl)phenyl)-3-methoxyacrylate **3a** was introduced into the batches. The reaction temperature was raised to 80 °C and maintained for 2 h; the reaction was monitored by TLC. Afterward, the reaction mixture was poured into water and extracted with ethyl acetate (50 mL). The organic phase was washed successively with water (20 mL) and saturated brine (20 mL), then dried, filtered, and evaporated under reduced pressure. The residue was purified by silica gel chromatography using ethyl acetate/petroleum ether (1:10, *v/v*) as the eluent in a temperature range of 60–90 °C to obtain 4.4 g of the compound **4b** as a yellow oil at a yield of 87%. ^1^H NMR (500 MHz, DMSO-*d*_6_) δ 7.61 (s, 1H, CH), 7.48–7.50 (m, 1H, Ar-H), 7.22–7.36 (m, 2H, Ar-H), 7.13–7.15 (m, 1H, Ar-H), 7.11 (s, 1H, pyrimidy1-H), 5.35 (s, 2H, CH_2_), 3.78 (s, 3H, CH_3_), 3.57 (s, 3H, CH_3_), 3.33 (t, *J* = 7.0 Hz, 2H, CH_2_), 2.73–2.81 (m, 2H, CH_2_); ^13^C NMR (125 MHz, DMSO-*d*_6_) δ 171.76, 169.42, 166.85, 160.87, 160.81, 154.89 (q, *J* = 36.3 Hz), 153.24 (m), 134.29, 132.65, 131.30, 128.64, 128.14, 127.62, 127.81 (m), 120.24 (q, *J* = 272.5 Hz), 108.66, 101.43(q, *J* = 2.5 Hz), 67.12, 61.79, 51.16, 25.49, 25.35 (dd, *J*_1_ = 21.3 Hz, *J*_2_ = 2.5 Hz); HRMS: m/z 531.0787 (M + Na)^+^(calcd. [M + Na]^+^ 531.0784.

### 3.3. Acaricidal Activities Assay

The detailed procedure of the acaricidal activity against adult *T. cinnabarinus* was measured according to the related literature [51].

Each of the test compounds was first dissolved in a mixture of acetone and water (at a volume ratio of 9 to 1) containing 0.1% TWEEN^®^ 80 to furnish the stock solutions. A series of test solutions were then prepared by diluting the stock solutions with water containing 0.1% TWEEN^®^ 80. Afterward, the kidney bean plants with one true leaf were infested with *T. cinnabarinus* (carmine spider mite) prior to spraying. An airbrush was used to spray the plants with the test solutions, and each bioassay was replicated three times at a temperature of 25 ± 1 °C to meet the statistical requirements. After the plants were dried, they were transferred to a maintenance room for observation. The mortality rate of the spider mite was investigated after 72 h. The mites that did not either fly away or respond to the touch of a fine brush were considered to be dead. The rate of mortality was evaluated according to a percentage scale of 0–100, in which 0 indicates no activity of the test solutions against the spider mites, and 100 indicates totally killing them. When the percentage of the mortality of the blank control was less than 5%, the results of the treatment were directly used. However, if the percentage of the mortality of the blank control ranged from 5 to 20%, the results were corrected by the means of the following equation:

*V* = (*X* − *Y*)/*Y* × 100 (1)

where, *V* is the value of the corrected percentage of mortality, and *X* represents the viability of the blank control; *Y* stands for the viability of the treatment. As a positive control, commercial fluacrypyrim was also evaluated using exactly the same procedure.

## 4. Conclusions

In summary, a series of phenyl methoxyacrylates containing 2-alkenylthiopyrimidine derivatives were designed, synthesized, and evaluated in terms of acaricidal activity. Compound **4j** exhibited significant acaricidal activity against the adults, larvae, and eggs of *T. cinnabarinus* (Boisduval), significantly higher than fluacrypyrim. Furthermore, the field trials proved that the acaricidal efficacy of compound **4j** was approximately equivalent to those of cyetpyrafen and cyenopyrafen. The field trials also demonstrated that compound **4j** at a concentration of 100 mg L^−1^ presented a control effect of 96.25% and 81.02% against *Panonychuscitri* after 10 and 20 days of treatment, respectively, indicating that it has long-lasting persistence (20 days) in field trials. It also acts rapidly against *Panonychuscitri* by reaching a controlling effect of 96.61% after 3 days of treatment. The compound **4j** has quite low acute toxicity to mammals, birds, and honeybees. Finally, the findings of the current work suggested that the compound **4j** (3,3-dichloroallylthio, trifluoromethyl, pyrimidine) could be a novel acaricide candidate for the control of spider mites, which is worthy of being further studied.

## Figures and Tables

**Table 1:** The physical properties of the intermediates **1a**–**1f** and **2a**–**2o.**

**Compd.**	**R_1_**		**Appearance**	**m.p./°C**	**Yield/%**
**1a**	CHF_2_		Light yellow solid	281.3–282.4	86
**1b**	CF_3_		Light yellow solid	247.5–248.2	90
**1c**	CH_3_		White solid	>300	90
**1d**	C_2_H_5_		White solid	226.2–228	87
**1e**	*n*-Pr		White solid	222.1–222.9	81
**1f**	*c*-Pr		White solid	241.1–243.5	82
**Compd.**	**R_1_**	**R_2_**	**Appearance**	**m.p./°C**	**Yield/%**
**2a**	CHF_2_	4,4-difluorobut-3-enyl	Light yellow solid	79.6–80.5	89
**2b**	CHF_2_	allyl	Yellow solid	112.0–114.7	84
**2c**	CHF_2_	2-chloroallyl	Light yellow solid	103.7–104.3	79
**2d**	CHF_2_	3-chloroallyl	Brown solid	106.8	84
**2e**	CHF_2_	3,3-dichloroallyl	Brown solid	108.6	87
**2f**	CF_3_	4,4-difluorobut-3-enyl	Light yellow solid	82.6–83.7	92
**2g**	CF_3_	allyl	Light yellow solid	132.9–133.6	82
**2h**	CF_3_	2-chloroallyl	Light yellow solid	116.2–116.6	93
**2i**	CF_3_	3-chloroallyl	Light yellow solid	103	83
**2j**	CF_3_	3,3-dichloroallyl	Light yellow solid	110–111.4	87
**2k**	CF_3_	3-methylbut-2-enyl	White solid	132.7–133.5	80
**2l**	CH_3_	3,3-dichloroallyl	White solid	174.1–174.7	85
**2m**	C_2_H_5_	3,3-dichloroallyl	White solid	118.9–120.2	84
**2n**	*n*-Pr	3,3-dichloroallyl	White solid	115.4–117.3	81
**2o**	*c*-Pr	3,3-dichloroallyl	White solid	174.3	82

**Table 2:** The physical properties of the compounds **4a**–**4u.**

Compd.	R_1_	R_2_	A	Appearance/m.p./°C	Yield/%
**4a**	CHF_2_	4,4-difluorobut-3-enyl	CH	Yellow oil	84
**4b**	CF_3_	4,4-difluorobut-3-enyl	CH	Yellow oil	87
**4c**	CHF_2_	allyl	CH	White solid, 59.6–60.3 °C	82
**4d**	CHF_2_	2-chloroallyl	CH	Colorless oil	77
**4e**	CHF_2_	3-chloroallyl	CH	Yellow oil	76
**4f**	CHF_2_	3,3-dichloroallyl	CH	Yellow oil	80
**4g**	CF_3_	allyl	CH	White solid, 63.4–64.8 °C	82
**4h**	CF_3_	2-chloroallyl	CH	White solid, 73.8–74.9 °C	79
**4i**	CF_3_	3-chloroallyl	CH	Colorless oil,	77
**4j**	CF_3_	3,3-dichloroallyl	CH	White solid, 57.4–58.3 °C	83
**4k**	CF_3_	3-methylbut-2-enyl	CH	Yellow oil	75
**4l**	CHF_2_	allyl	N	Yellow oil	82
**4m**	CHF_2_	3-chloroallyl	N	Colorless oil	88
**4n**	CHF_2_	3,3-dichloroallyl	N	White solid, 65.4–65.6 °C	81
**4o**	CF_3_	allyl	N	Yellow oil	80
**4p**	CF_3_	3-chloroallyl	N	Colorless oil	78
**4q**	CF_3_	3,3-dichloroallyl	N	White solid, 76.4–77.5 °C	83
**4r**	CH_3_	3,3-dichloroallyl	CH	Yellow oil	83
**4s**	C_2_H_5_	3,3-dichloroallyl	CH	Yellow oil	80
**4t**	*n*-Pr	3,3-dichloroallyl	CH	Yellow oil	76
**4u**	*cyc*-Pr	3,3-dichloroallyl	CH	Colorless oil	81

**Table 3:** Acaricidal activities of the compounds **4a**–**4u** against the adults of *T. cinnabarinus*.

Compd.	Activities (%) against Adults at Concentration ^a^ (mg L^−1^)				Compd.	Activities (%) against Adults at Concentration ^a^ (mg L^−1^)			
	500	100	20	4		500	100	20	4
**4a**	90 ± 6	20 ± 10	0	0	**4l**	0	//	//	//
**4b**	0	0	//	//	**4m**	0	//	//	//
**4c**	100 ± 0	80 ± 3	80 ± 2	20 ± 6	**4n**	0	//	//	//
**4d**	90 ± 8	100 ± 0	50 ± 3	0	**4o**	100 ± 0	60 ± 6	30 ± 4	30 ± 7
**4e**	100 ± 0	80 ± 2	20 ± 7	0	**4p**	0	//	//	//
**4f**	100 ± 0	80 ± 4	80 ± 5	20 ± 7	**4q**	30 ± 8	//	//	//
**4g**	100 ± 0	50 ± 7	0	0	**4r**	0	//	//	//
**4h**	100 ± 0	90 ± 5	50 ± 6	0	**4s**	0	//	//	//
**4i**	100 ± 0	100 ± 0	80 ± 10	70 ± 7	**4t**	0	//	//	//
**4j**	100 ± 0	100 ± 0	80 ± 2	80 ± 4	**4u**	0	//	//	//
**4k**	100 ± 0	100 ± 0	80 ± 3	0	**fluacrypyrim**	100 ± 0	100 ± 0	80 ± 5	40 ± 7

^a^ refers to not tested.

**Table 4:** LC_50_ of the acaricidal activity against *T. cinnabarinus.*

Compd.	Adults		Larvae ^a^		Ovicidal	
	LC_50_ (mg L^−1^)	95% Confidence Interval	LC_50_ (mg L^−1^)	95% Confidence Interval	LC_50_ (mg L^−1^)	95% Confidence Interval
**4i**	138.626	122.279–158.246	-	-	146.465	115.367–189.178
**4j**	5.324	3.209–7.375	1.163	1.037–1.302	17.183	13.903–21.257
Fluacrypyrim	4.178	1.203–7.112	2.009	1.777–2.297	43.332	32.444–63.118

^a^ refers to not tested.

**Table 5:** Acaricidal activity of the compound **4j** against *P. Citri* in field tests (Nanning, China).

Compd.	Concentration (mg L^−1^)	Effect on Controlling *Panonychuscitri*(%)							
		3 days	sig.	7 days	sig.	10 days	sig.	20 days	sig.
**98% 4j**	100	96.61	a	97.36	b	96.25	a	81.02	a
cyetpyrafen	100	97.70	a	100.00	a	99.38	a	89.79	a
cyenopyrafen	100	92.26	a	97.85	b	96.43	a	80.87	b

The significance level is 0.05.

**Table 6:** Toxicity results of the compound **4j**.

Acute Toxicity Test	Species	Results
Oral LD_50_ (mg/kg)	Rat	>500 (female)
Oral LD_50_ (mg a.i./kg)	*Coturnix japonica*	>2090
Oral 48 h LD_50_ (µg a.i./honeybee)	*Apismellifera L.*	>116
Eye irritation	Rabbit	Moderate irritation

## Supplementary Materials

The following are available online, the data of title compounds **4a**–**4u** and spectrogram of title compounds **4a**–**4u** (Figures S1–S36) are presented as Supporting Information.

## References

[1] Chen Q.S., Zhao S., Zou J., Shi L., He L. (2012). Monitoring of acaricide resistance in *Tetranychus Cinnabarinus*. Chin. J. Appl. Entomol..

[2] Ma L.N., Hu J.H., Lei H.D. (2008). Research progress of botanical acaricides. Chin. Agric. Sci. Bull..

[3] Hua N.Z. (2016). A review of new high efficiency and low toxicity acaricides. World Pestic..

[4] Van Leeuwen T., Vontas J., Tsagkarakou A., Dermauw W., Tirry L. (2010). Acaricide resistance mechanisms in the two-spotted spider mite *Tetranychusurticae* and other important acari: A review. Insect Biochem. Mol. Biol..

[5] Ouyang Y.L., Montez G.H., Liu L., Grafton-Cardwella E.E. (2012). Spirodiclofen and spirotetramat bioassays for monitoring resistance in citrus red mite panonychus citri (Acari: Tetranychidae). Pest Manage. Sci..

[6] Liang P., Shen J.Z., You W.L. (1999). Research on advances of acaricide mechanism. Pestic. Sci. Adm..

[7] Mark A.D. (2005). Review acaricide mode of action. Pest Manag. Sci..

[8] Van Leeuwen T., Tirry L., Yamamoto A., Nauen R., Wannes D. (2015). The economic importance of acaricides in the control of phytophagous mites and an update on recent acaricide mode of action research. Pestic. Biochem. Physiol..

[9] Khalighi M., Dermauw W., Wybouw N., Bajda S., Osakabe M., Tirrya L., Van Leeuwena T. (2016). Molecular analysis of cyenopyrafen resistance in the two-spotted spider mite *Tetranychus*. Pest Manag. Sci..

[10] Kramer T., Nauen R. (2011). Monitoring of spirodiclofen susceptibility in field populations of European red mites, *Panonychusulmi* (Koch) (Acari: *Tetranychidae*), and the cross-resistance pattern of a laboratory-selected strain. Pest Manag. Sci..

[11] Feng K.Y., Yang Y.W., Wen X., Ou S.Y., Zhang P., Yu Q., Zhang Y.C., Shen G.M., Xu Z.F., Li J.H. (2019). Stability of cyflumetofen resistance in *Tetranychus Cinnabarinus* and its correlation with glutathione-*S*-transferase gene expression. Pest Manag. Sci..

[12] Sun X.T., Liu J.L., Song Y.Q., Liu S.W. Research progress on strobilurin acaricides. Proceedings of the 14th Annual Meeting of the Pesticide Committee of the Chinese Chemical Society.

[13] Liu C.L., Guan A.Y., Liu Z.L. (2002). The novel (*E*)-methyl-*β*-methoxyacrylate acaricides fluacrypyrim. Word Pestic..

[14] Beautement K., Clough J.M., de Fraine P.J., Godfrey C.R.A. (1991). Fungicidal beta-methoxyacrylates: From natural products to novel synthetic agricultural fungicides. Pestic. Sci..

[15] Chai B.S., Liu C.L., Li H.C., Zhang H., Liu S.W., Huang G., Chang J.B. (2011). The discovery of SYP-10913 and SYP-11277: Novel strobilurin acaricides. Pest Manag. Sci..

[16] Liu A.P., Wang X.G., Chen C., Pei H., Mao C.H., Wang Y.J., He H.J., Huang L., Liu X.P., Hu Z.B. (2009). The discovery of HNPC-A3066: A novel strobilurin acaricide. Pest Manag. Sci..

[17] Liu C.L., Yang J.C. (2018). Handbook of Modern Pesticide.

[18] D’Amico J. (1971). Certain 2-(Haloalkenylthio)-4,6-dimethylpyrimidines. U.S. Parent.

[19] Tetsuo T., Yasutomo T., Mitsuaki T., Seiji T., Hiroshi H. (1987). Pyrimidinyloxyalkanamide Derivatives and Herbicide Compositions Containing Them. European Patent.

[20] Edwards L.H. (1983). Fungicidal and Insecticidal 2-Thiohaloalkenyl-4-dialkoxyphosphino-thioyloxy-6-alkyl-1,3-pyrimidines. U.S. Patent.

[21] Katsumi M., Ikumi U., Tsuyoshi A., Katsumi F., Yoshiyuki K., Norimichi M. (1998). Preparation of Pyrimidinyloxyalkanoic Amide Derivatives as Fungicides for Agricultural and Horticultural Use. World Patent.

[22] Fitzjohn S., Robinson M.P., Turnbull M.D. (1994). Heterocyclic Compounds with Parasitical Activity. World Patent.

[23] Turnbull M.D. (1992). Nematicide Pyrimidine Derivatives. European Patent.

[24] Ilardi E.A., Vitaku E., Njardarson J.T. (2014). Data-mining for sulfur and fluorine: An evaluation of pharmaceuticals to reveal opportunities for drug design and discovery. J. Med. Chem..

[25] Feng M., Tang B., Liang S., Jiang X. (2016). Sulfur containing scaffolds in drugs: Synthesis and application in medicinal chemistry. Curr. Top. Med. Chem..

[26] Smith B.R., Eastman C.M., Njardarson J.T. (2014). Beyond C,H,O, and N analysis of the elemental composition of U.S. FDA approved drug architectures. J. Med. Chem..

[27] Xiong H.Y., Pannecoucke X., Besset T. (2016). Recent advances in the synthesis of SCF_2_H- and SCF_2_FG-containing molecules. Chem. Eur. J..

[28] Mishra A., Ma C.Q., Baüerle P. (2009). Functional oligothiophenes: Molecular design for multidimensional nanoarchitectures and their applications. Chem. Rev..

[29] Scott K.A., Njardarson J.T. (2018). Analysis of US FDA-approved drugs containing sulfur atoms. Top. Curr. Chem..

[30] Wang M., Fan Q., Jiang X. (2016). Transition-metal-free diarylannulated sulfide and selenide construction via radical/anion-mediated sulfur−iodine and selenium−iodine exchange. Org. Lett..

[31] Borthwick A.D. (2012). 2,5-Diketopiperazines: Synthesis, reactions, medicinal chemistry, and bioactive natural products. Chem. Rev..

[32] Nicolaou K.C., Hale C.R.H., Nilewski C., Ioannidou H.A. (2012). Constructing molecular complexity and diversity: Total synthesis of natural products of biological and medicinal importance. Chem. Soc. Rev..

[33] Liu H., Jiang X. (2013). Transfer of sulfur: From simple to diverse. Chem. Asian J..

[34] Davison E.K., Sperry J. (2017). Natural products with heteroatom-rich ring systems. J. Nat. Prod..

[35] http://www.alanwood.net/pesticides/index.html.

[36] Liu C.L., Chai B.S. (2013). New Agrochemicals Discovery and Synthesis.

[37] Lin Y., Zhang S.Z., Block E., Katz L.C. (2005). Encoding social signals in the mouse main olfactory bulb. Nature.

[38] Nair D.P., Podgórski M., Shunsuke C., Gong T., Xi W.X., Fenoli C.R., Bowman C.N. (2014). The thiol-Michael Addition Click Reaction: A powerful and widely used tool in materials chemistry. Chem. Mater..

[39] Bürchstümmer H., Weissenstein A., Bilalas D., Würthner F. (2011). Synthesis and characterization of optical and redox properties of bithiophene-functionalized diketopyrrolopyrrole chromophores. J. Org. Chem..

[40] Rakhimov A.I., Titova E.S., Fedunov R.G., Babkin V.A. (2008). Special features of the nucleophilic substitution of halogen in alkyl and benzyl halides with anions generated from 4-hydroxy-2-mercapto-6-methylpyrimidine. Chem. Heterocycl. Compd..

[41] Harold W.B., Irving G., Karl D. (1948). The synthesis of 5-halogeno-2-thiouracil and 6-methyl-5-halogeno-2-thiouracil derivatives. J. Am. Chem. Soc..

[42] Uneyama K. (2006). Organofluorine Chemistry.

[43] Kirsch P. (2013). Modern Fluoroorganic Chemistry: Synthesis, Reactivity, Applications.

[44] Erkin A.V., Krutikov V.I., Chubraev M.A. (2004). Synthesis of 2-(pyrazol-1-yl)pyrimidine derivatives by cyclocondensation of ethyl acetoacetate (6-methyl-4-oxo-3,4-dihydropyrimidin-2-yl)hydrazone with aromatic aldehydes. Russ. J. Gen. Chem..

[45] Tang M.P., Jia J.X., Xue J.J., Liu J.P. (2017). Action of thiouracil derivatives as novel thermal stabilizers for poly (vinyl chloride) and the synergistic effect with calcium stearate. Russ. J. Appl. Chem..

[46] Slivka N.Y., Gevaza Y.I., Staninets V.I. (2004). Halocyclization of substituted 2-(alkenylthio)pyrimidin-6-ones. Chem. Heterocycl. Compd..

[47] Yang Z.Y., Hao S.L. (2014). Efficient Green Synthesis Method of Agricultural Fungicide Picoxystrobin. Chinese Patent.

[48] Yasuyuki M., Takahiro S., Yutaka I., Hiroyuki Y., Makoto F., Mitsuru T., Yoshiyuki I., Satoru Y., Noriaki K. (2004). Process for Producing Acrylic Acid Derivative. U.S. Parent.

[49] Bernd W., Siegbert B., Franz S., Thomas K., Franz R., Eberhard A., Gisela L. (1996). Preparation of (pyridinyloxy)phenylglyoxylate O-methyl Oximes as Agrochemical Fungicides. U.S. Parent.

[50] Sanghyuck L., Oh Seok K., Chang-Soo L., Won M., Ban H.S., Ra C.S. (2017). Synthesis and biological evaluation of kresoxim-methyl analogues as novel inhibitors of hypoxia-inducible factor (HIF)-1 accumulation in cancer cells. Bioorg. Med. Chem. Lett..

[51] Xie Y., Xu Y., Liu C.L., Guan A.Y., Ban L.F., Ding F., Peng W. (2017). Intermediate derivatization method in the discovery of new acaricide candidate: Synthesis of *N*-substituted piperazines derivatives and their activity against phytophagous mites. Pest Manag. Sci..

