# Supplementary material for: Design, Synthesis, and Acaricidal Activity of Phenyl Methoxyacrylates Containing 2-Alkenylthiopyrimidine
Source: Molecules. 2020 Jul 25;25(15):3379. doi: 10.3390/molecules25153379 (PMC7435930; doi:10.3390/molecules25153379)
Supplement: Supplementary file 1 [file molecules-25-03379-s001.pdf]

Supplementary Materials

Design, Synthesis, and Acaricidal Activity of Phenyl Methoxyacrylates Containing 2-Alkenylthiopyrimidine

Shulin Hao *, Zengfei Cai, Yangyang Cao and Xiaohua Du *

Zhejiang University of Technology, Catalytic Hydrogenation Research Center, Zhejiang Key Laboratory of Green Pesticides and Cleaner Production Technology, Zhejiang Green Pesticide Collaborative Innovation Center, Hangzhou 310014, China; caizengfei@zjut.edu.cn (Z.C.); yycao@zjut.edu.cn (Y.C.)

* Correspondence: shulin0525@163.com (S.H.); duxiaohua@zjut.edu.cn (X.D.); Tel.: +86-571-88320430 (X.D.)

Received: 19 June 2020; Accepted: 24 July 2020; Published: 25 July 2020

Supporting Information

1. The data of title compounds 4a-4u……………………………………………………………...2
2. Spectrogram of title compounds 4a-4u (Fig. S1–Fig. S36) ………………………………………...7

1.The data of title compounds 4a–4u.

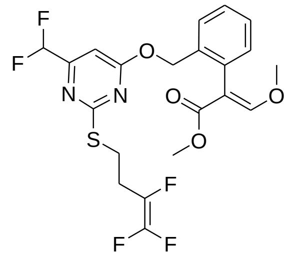

Data for

(*E*)-methyl 2-(2-((6-(difluoromethyl)-2-(3,4,4-trifluorobut-3-enylthio)pyrimidin-4-yloxy)methyl)phenyl)-3-methoxyacrylate (**4a**). Yellow oil, yield, 84%. ^{1}H NMR (500 MHz, DMSO-d_6) δ 7.63 (s, 1H, CH), 7.47-7.49 (m, 1H, Ar-H), 7.32-7.35 (m, 2H, Ar-H), 7.14 (dd, *J* = 7.5 Hz, *J* = 4.5 Hz, 1H, Ar=H), 6.83(s, 1H, pyrimidy1-H), 6.84 (t, *J* = 65.0 Hz, 1H, CHF_2), 5.23 (s, 2H, CH_2), 3.79 (s, 3H, CH_3), 3.58 (s, 3H, CH_3), 3.32 (t, *J*=8.5 Hz, 2H, CH_2), 2.73-2.81 (m, 2H, CH_2). ^{13}C NMR (125 MHz, DMSO-d_6) δ 170.80, 169.16, 166.80, 160.69 (t, *J* = 43.8 Hz), 160.66, 153.22 (m), 134.47, 132.60, 131.23, 128.48, 127.99, 127.80 (m), 127.53, 111.77 (t, *J* =298.8 Hz), 108.71, 100.68 (t, *J* =5.0 Hz), 66.68, 61.72, 51.09, 26.30, 25.38, 25.28 (dd, *J* = 26.3, 2.5 Hz), 25.16. HRMS m/z 513.0877 [M + Na]$^+$ (calcd [M + Na]$^+$ 513.0878).

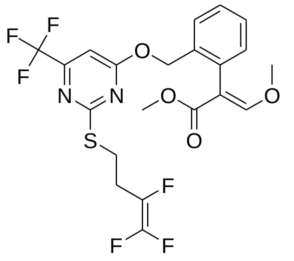

Data for

(*E*)-methyl 3-methoxy-2-(2-((2-(3,4,4-trifluorobut-3-enylthio)- 6-(trifluoromethyl)pyrimidin-4-yloxy)methyl)phenyl)acrylate (**4b**). Yellow oil, yield, 87%. ^{1}H NMR (500 MHz, DMSO-d_6) δ 7.61 (s, 1H, CH), 7.48-7.50 (m, 1H, Ar-H), 7.22-7.36 (m, 1H, Ar-H), 7.13-7.15 (m, 1H, Ar-H), 7.11 (s, 1H, pyrimidy1-H), 5.35 (s, 2H, CH_2), 3.78 (s, 3H, CH_3), 3.57 (s, 3H, CH_3), 3.33 (t, *J* =7.0 Hz, 2H, CH_2), 2.73-2.81 (m, 2H, CH_2); ^{13}C NMR (125 MHz, DMSO-d_6) δ 171.76, 169.42, 166.85, 160.87, 160.81, 154.89 (q, *J* = 36.25 Hz), 153.24 (m), 134.29, 132.65, 131.30, 128.64, 128.14, 127.62, 127.81 (m), 120.24 (q, *J* =272.5 Hz), 108.66, 101.43 (q, *J* = 2.5 Hz), 67.12, 61.79, 51.16, 25.49, 25.35 (dd, J_1 = 21.3 Hz, J_2 =2.5 Hz); HRMS: m/z 531.0787 (M + Na)$^+$(calcd. [M + Na]$^+$ 531.0784.

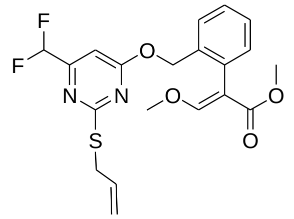

Data for

(*E*)-methyl 2-(2-((2-(allylthio)-6-(difluoromethyl)pyrimidin-4-yloxy)methyl)phenyl)-3-methoxyacrylate (**4c**). White solid, yield, 82%, m.p., 59.6-60.3 °C. ^{1}H NMR (400 MHz, $CDCl_3$) δ 7.56 (s, 1H), 7.48-7.50 (m, 1H, Ar-H), 7.34-7.36 (m, 2H, Ar-H), 7.18-7.20 (m, 1H, Ar-H), 6.63(s, 1H, pyrimidy1-H), 6.38 (t, *J*=68.0 Hz, 1H, CHF_2), 5.91-6.01 (m, 1H, CH), 5.35 (s, 2H, CH_2), 5.30 (d, *J* = 17.2 Hz, 1H, 0.5*CH_2), 5.13 (d, *J* = 10.0 Hz, 1H, 0.5*CH_2), 3.79 (d, *J* = 6.0 Hz, 2H, CH_2), 3.79 (s, 3H, CH_3).3.67 (s, 3H, CH_3).

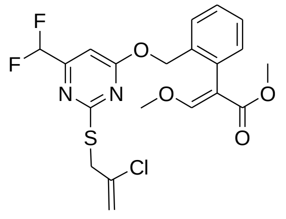

Data for

(*E*)-methyl 2-(2-((2-(2-chloroallylthio)-6-(difluoromethyl)pyrimidin-4-yloxy)methyl)phenyl)-3-methoxyacrylate (4**d)**. Colorless oil, yield, 77%. ^{1}H NMR (400 MHz, $CDCl_3$) δ 7.56 (s, 1H), 7.48-7.50 (m, 1H, Ar-H), 7.34-7.36 (m, 2H, Ar-H), 7.17-7.24 (m, 1H, Ar-H), 6.65 (s, 1H, pyrimidy1-H), 6.38 (t, *J* = 54.8 Hz, 1H, CHF_2), 5.52 (s, 1H, CH_2) , 5.35 (s, 2H, CH_2), 5.29 (s, 1H, CH_2), 4.06 (s, 2H, CH_2), 3.80 (s, 3H, CH_3), 3.67 (s, 3H, CH_3).

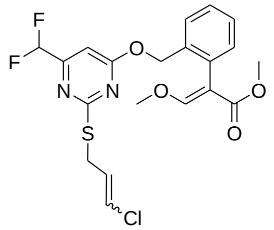

Data for

(*E*)-methyl 2-(2-((2-((Z/*E*)-3-chloroallylthio)-6-(difluoromethyl)pyrimidin-4-yloxy)methyl)phenyl)-3-methoxyacrylate (**4e**). Yellow oil, yield, 76%. ^{1}H NMR (400 MHz, $CDCl_3$) δ 7.62 (s, 0.66*1H, CH), 7.61 (s, 0.34*1H, CH), 7.45-7.49 (m, 1H, Ar-H), 7.33-7.36 (m, 2H, Ar-H), 7.18-7.20 (m, 1H, Ar-H), 6.38 (t, *J* = 44.0 Hz, 0.66*1H), 6.209 (t, *J* = 44.0 Hz, 0.34*1H), 6.66 (s, 0.66*1H, pyrimidy1-H), 6.65 (s, 0.34*1H, pyrimidy1-H), 5.98-6.05 (m, 1H), 5.35 (s, 2H, CH_2), 3.93 (d, *J* = 10.0 Hz, 0.34*2H, CH_2), 3.75 (d, *J* = 10.0 Hz, 0.66* 2H, CH_2), 3.80 (s, 0.66*3H, CH_3), 3.79 (s, 0.34*3H, CH_3), 3.68 (s, 0.66*3H, CH_3), 3.67 (s, 0.34*3H, CH_3). HRMS m/z 457.0793 $[M + H]^+$(calcd $[M + H]^+$ 457.0795).

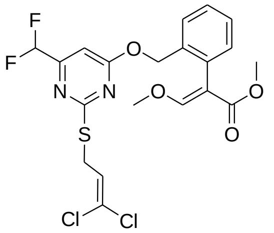

Data for

(*E*)-methyl 2-(2-((2-(3,3-dichloroallylthio)-6-(difluoromethyl)pyrimidin-4-yloxy)methyl)phenyl)-3-methoxyacrylate (**4f**). Yellow oil, yield, 80%. ^{1}H NMR (500 MHz, $CDCl_3$) δ 7.60 (s, 1H, CH), 7.49-7.51 (m, 1H, Ar-H), 7.37-7.40 (m, 2H, Ar-H), 7.21-7.23 (m, 1H, Ar-H), 6.70 (s, 1H, pyrimidy1-H), 6.43 (t, *J* =55.0 Hz, 1H, CHF_2), 6.11 (t, *J* = 7.5 Hz, 1H, CH), 5.38 (s, 2H, CH_2), 3.88 (d, *J* = 7.5 Hz, 2H, CH_2), 3.84 (s, 3H, CH_3), 3.71 (s, 3H, CH_3).

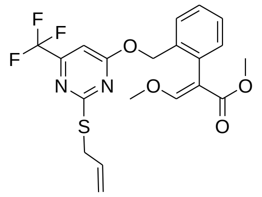

Data for

(*E*)-methyl 2-(2-((2-(allylthio)-6-(trifluoromethyl)pyrimidin-4-yloxy)methyl)phenyl)-3-methoxyacrylate (**4g**). White solid, yield, 82%. m.p., 63.4-64.8 °C. ^{1}H NMR (400 MHz, $CDCl_3$) δ 7.56 (s, 1H), 7.46-7.50 (m, 1H, Ar-H), 7.33-7.38 (m, 2H, Ar-H), 7.18-7.20 (m, 1H, Ar-H), 6.67(s, 1H, pyrimidy1-H), 5.90-6.00(m, 1H, CH), 5.36 (s, 2H, CH_2), 5.35 (d, *J* = 16.8 Hz, 1H, 0.5*CH_2), 5.14 (d, *J* = 10.0 Hz, 1H, 0.5*CH_2), 3.80 (s, 3H, CH_3), 3.79 (d, *J* = 6.0 Hz, 2H, CH_2), 3.68 (s, 3H, CH_3). ^{13}C NMR (100 MHz, $CDCl_3$) δ 172.87, 169.43, 167.72, 160.15, 156.03 (q, *J* = 35.5 Hz), 134.36, 133.05, 132.26, 131.32, 128.79, 128.37, 128.11, 120.34 (q, *J* = 273.3 Hz), 118.28, 110.01, 100.76 (q, *J* = 3.2 Hz), 67.18, 62.05, 51.72, 33.86. HRMS m/z 463.0913 $[M + Na]^+$(calcd $[M + Na]^+$ 463.0910).

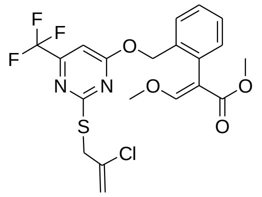

Data for

(*E*)-methyl 2-(2-((2-(2-chloroallylthio)-6-(trifluoromethyl)pyrimidin-4-yloxy)methyl)phenyl)-3-methoxyacrylate (**4h**). White solid, yield, 79%. m.p., 73.8-74.9 °C. ^{1}H NMR (400 MHz, $CDCl_3$) δ 7.57 (s, 1H), 7.48-7.50 (m, 1H, Ar-H), 7.35-7.37 (m, 2H, Ar-H), 7.18-7.20 (m, 1H, Ar-H), 6.69 (s, 1H, pyrimidy1-H), 5.56 (s, 1H, 0.5*CH_2) , 5.36 (s, 2H, CH_2), 5.31 (s, 1H, 0.5*CH_2), 4.07 (s, 2H, CH_2), 3.82 (s, 3H, CH_3), 3.68 (s, 3H, CH_3).

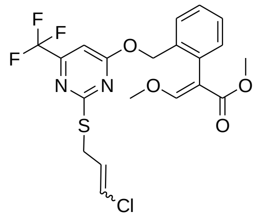

Data for

(*E*)-methyl 2-(2-((2-((Z/*E*)-3-chloroallylthio)-6-(trifluoromethyl)pyrimidin-4-yloxy)methyl)phenyl)-3-methoxyacrylate (**4i**). Colorless oil, yield, 77%. ^{1}H NMR (500 MHz, $CDCl_3$) δ 7.66 (s, 0.66*1H, CH), 7.65 (s, 0.34*1H, CH), 7.48-7.52 (m, 1H, Ar-H), 7.33-7.37 (m, 2H, Ar-H), 7.14-7.17 (m, 1H, Ar-H), 7.12 (s, 0.66*1H, pyrimidy1-H), 7.12 (s, 0.34*1H, pyrimidy1-H), 6.45-6.51 (m, 1H), 6.05-6.16 (m, 1H, CH), 5.37 (s, 2H, CH_2), 3.94 (d, *J* = 10.0 Hz,

0.34*2H, CH_2), 3.85 (d, J = 10.0 Hz, 0.66* 2H, CH_2), 3.81 (s, 0.66*3H, CH_3), 3.80 (s, 0.34*3H, CH_3), 3.59 (s, 0.66*3H, CH_3), 3.59 (s, 0.34*3H, CH_3).

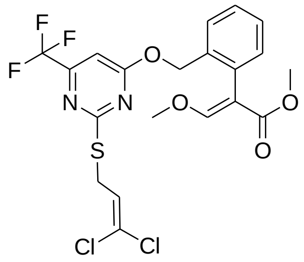

Data for
(*E*)-methyl 2-(2-((2-(3,3-dichloroallylthio)-6-(trifluoromethyl)pyrimidin-4-yloxy)methyl)phenyl)-3-methoxyacrylate (**4j**). White solid, yield, 83%. m.p., 57.4-58.3 °C. ^{1}H NMR (500 MHz, $CDCl_3$) δ 7.62 (s, 1H, CH), 7.51-7.52 (m, 1H, Ar-H), 7.38-7.42 (m, 2H, Ar-H), 7.23-7.25 (m, 1H, Ar-H), 6.76 (s, 1H, pyrimidy1-H), 6.16 (t, J = 10.0 Hz, 1H, CH), 5.42 (s, 2H, CH_2), 3.90 (d, J = 10.0 Hz, 2H, CH_2), 3.85 (s, 3H, CH_3), 3.73 (s, 3H, CH_3). ^{13}C NMR (125 MHz, $CDCl_3$) δ 172.29, 169.59, 167.67, 160.16, 156.14 (q, J = 35.8 Hz), 134.24, 132.23, 131.39, 128.65, 128.41, 128.11, 125.38, 120.26 (q, J = 272.8 Hz), 110.02, 101.17 (q, J=3.0 Hz), 67.72, 62.03, 51.68, 29.68. ^{19}F NMR (470 MHz, $CDCl_3$) δ -70.48. HRMS m/z 509.0313 $[M + H]^+$(calcd $[M + H]^+$ 509.0311).

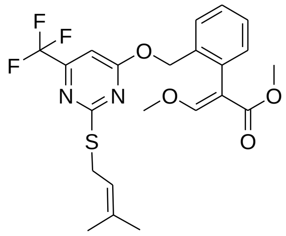

Data for
(*E*)-methyl 3-methoxy-2-(2-((2-(3-methylbut-2-enylthio)-6-(trifluoromethyl)pyrimidin-4-yloxy)methyl)phenyl)acrylate (**4k**). Yellow oil, yield, 75%. ^{1}H NMR (500 MHz, DMSO-d_6) δ 7.65 (s, H, CH), 7.50-7.52 (m, 1H, Ar-H), 7.34-7.36 (m, 2H, Ar-H), 7.14-7.16 (m, 1H, Ar-H), 7.06 (s, 1H, pyrimidy1-H), 5.37 (s, 2H, CH_2), 3.80 (d, 5H, CH_2, CH_3), 3.59 (s, 3H, CH_3), 1.71 (d, J = 5.0 Hz, 6H, $2CH_3$). HRMS m/z 469.1398 $[M + H]^+$(calcd $[M + H]^+$ 469.1403).

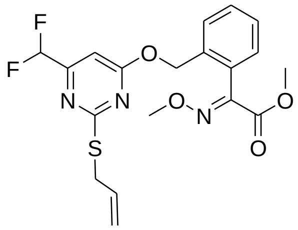

Data for
(*E*)-methyl 2-(2-((2-(allylthio)-6-(difluoromethyl)pyrimidin-4-yloxy)methyl)phenyl)-2-(methoxyimino)acetate (**4l**). Yellow oil, yield, 82%. ^{1}H NMR (400 MHz, $CDCl_3$) δ 7.49-7.51 (m, 1H, Ar-H), 7.40-7.46 (m, 2H, Ar-H), 7.20-7.22 (m, 1H, Ar-H), 6.61(s, 1H, pyrimidy1-H), 6.38 (t, J=68.0 Hz, 1H, CHF_2), 5.90-6.00 (m, 1H, CH), 5.33 (s, 2H, CH_2), 5.30 (d, J = 19.6 Hz, 1H, 0.5*CH_2), 5.14 (d, J = 10.0 Hz, 1H, 0.5*CH_2), 4.01 (s, 3H, CH_3), 3.85 (s, 3H, CH_3), 3.79 (d, J = 6.8 Hz, 2H, CH_2).

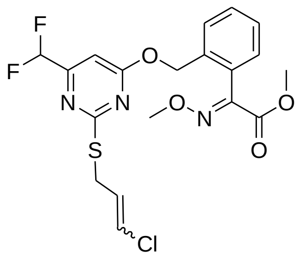

Data for
(*E*)-methyl 2-(2-((2-((Z/*E*)-3-chloroallylthio)-6-(difluoromethyl)pyrimidin-4-yloxy)methyl)phenyl)-2-(methoxyimino)acetate (**4m**). Colorless oil, yield, 88%. ^{1}H NMR (500 MHz, $CDCl_3$) δ 7.52 (d, J = 7.5 Hz, 1H, Ar-H), 7.42-7.47 (m, 2H, Ar-H), 7.24 (d, J = 7.5 Hz, 1H, Ar-H), 6.65 (s, 0.7*1H, pyrimidy1-H), 6.65 (s, 0.3*1H, pyrimidy1-H), 6.41 (t, J = 55.0 Hz, 1H, CHF_2), 6.26 (d, J = 13.0 Hz, 0.7*2H, CH_2), 6.18 (d, J = 7.0 Hz, 0.3*2H, CH_2), 6.02-6.07 (m, 1H), 5.35 (s, 2H, CH_2), 4.04 (s, 3H, CH_3), 3.87 (s, 3H, CH_3), 3.95 (d, J = 7.0 Hz, 0.3*2H, CH_2), 3.78 (d, J = 7.5 Hz, 0.7*2H, CH_2). ^{13}C NMR (125 MHz, $CDCl_3$) δ 171.86, 171.38, 169.24, 169.22, 163.23, 161.12 (t, J = 25.8 Hz), 161.04 (t, J = 25.8 Hz), 149.26, 133.79, 133.74, 130.16, 130.08, 129.59, 129.56, 128.92, 128.80, 128.71, 128.59, 128.42, 128.40, 127.34, 121.33, 121.02, 113.94,

113.85, 112.01, 111.92, 110.09, 109.99, 100.37 (t, *J* = 3.8 Hz), 100.14 (t, *J* = 3.75 Hz), 66.36, 66.33, 63.87, 30.93, 27.38. HRMS m/z 480.0567 [M + Na]$^+$(calcd [M + Na]$^+$ 480.0567).

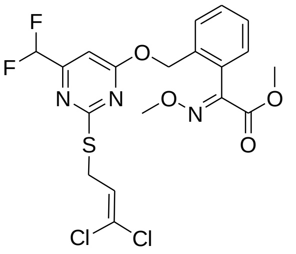

Data for

(*E*)-methyl 2-(2-((2-(3,3-dichloroallylthio)-6-(difluoromethyl)pyrimidin-4-yloxy)methyl)phenyl)-2-(methoxyimino)acetate (**4n**). White solid, yield, 81%, m.p., 65.4-65.6 °C. ^{1}H NMR (500 MHz, $CDCl_3$) δ 7.23-7.51 (m, 4H, Ar-H), 6.66 (s, 1H, pyrimidy1-H), 6.42 (t, *J* = 13.8 Hz, 1H), 6.10 (t, *J* = 1.5 Hz, 1H), 5.35 (s, 2H, CH_2), 4.04 (s, 3H, CH_3), 3.88 (s, 5H, CH_2 , CH_3). ^{13}C NMR (125 MHz, $CDCl_3$) δ 171.38, 169.27, 163.23, 161.16 (t, *J* = 25.0 Hz), 149.26, 133.70, 129.59, 128.84, 128.73, 128.45, 125.50, 123.66, 111.92 (t, *J* = 241.3 Hz), 100.40 (t, *J* = 3.8 Hz), 67.45, 63.89, 53.04, 29.62. HRMS m/z 492.0354 [M + H]$^+$(calcd [M + H]$^+$ 492.0358).

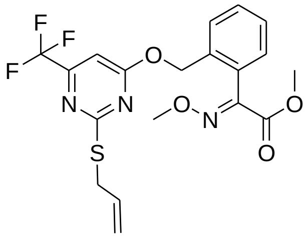

Data for

(*E*)-methyl 2-(2-((2-(allylthio)-6-(trifluoromethyl)pyrimidin-4-yloxy)methyl)phenyl)-2-(methoxyimino)acetate (**4o**). Yellow oil, yield, 77%. ^{1}H NMR (400 MHz, $CDCl_3$) δ 7.50-7.52 (m, 1H, Ar-H), 7.40-7.47 (m, 2H, Ar-H), 7.20-7.22 (m, 1H, Ar-H), 6.64(s, 1H, pyrimidy1-H), 5.90-6.00 (m, 1H, CH), 5.33 (s, 2H, CH_2), 5.32 (d, *J* = 16.8 Hz, 1H, 0.5*CH_2), 5.15 (d, *J* = 10.0 Hz, 1H, 0.5*CH_2), 4.02 (s, 3H, CH_3), 3.85 (s, 3H, CH_3), 3.80 (d, *J* = 6.8 Hz, 2H, CH_2). HRMS m/z 442.1046 [M + H]$^+$(calcd [M + H]$^+$442.1043).

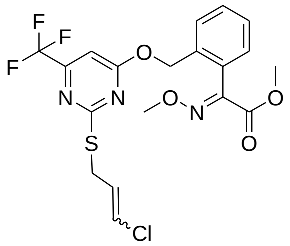

Data for

(*E*)-methyl 2-(2-((2-((Z/*E*)-3-chloroallylthio)-6-(trifluoromethyl)pyrimidin-4-yloxy)methyl)phenyl)-2-(methoxyimino)acetate (**4p**). Colorless oil, yield, 78%. ^{1}H NMR (400 MHz, $CDCl_3$) δ 7.49-7.51 (m, 1H, Ar-H), 7.41-7.47 (m, 2H, Ar-H), 7.20-7.22 (m, 1H, Ar-H), 6.67 (s, 0.6*1H, pyrimidy1-H), 6.66 (s, 0.4*1H, pyrimidy1-H), 6.29 (d, *J* = 13.2 Hz, 0.6*1H, CH_2), 6.17 (d, *J* = 6.8 Hz, 0. 4*1H, CH_2), 5.34 (d, 0. 4*2H, CH_2), 5.33 (d, 0.6* 2H, CH_2), 4.02 (s, 0.6*3H, CH_3), 4.02 (s, 0.4*3H, CH_3), 3.94 (d, *J* = 7.6 Hz, 0. 4*2H, CH_2), 3.86 (s, 3H, CH_3), 3.76 (d, *J* = 7.6 Hz, 0.6* 2H, CH_2). HRMS m/z 498.0474 [M + Na]$^+$(calcd [M + Na]$^+$ 498.0473).

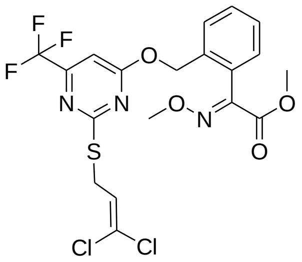

Data for

(*E*)-methyl 2-(2-((2-(3,3-dichloroallylthio)-6-(trifluoromethyl)pyrimidin-4-yloxy)methyl)phenyl)-2-(methoxyimino)acetate (**4q**). White solid, yield, 84%, m.p., 76.4-77.5 °C. ^{1}H NMR (500 MHz, $CDCl_3$) δ 7.53 (d, *J* = 7.0 Hz, 1H, Ar-H), 7.44-7.49 (m, 2H, Ar-H), 7.25 (d, *J* = 8.5 Hz ,1H, Ar-H), 6.70 (s, 1H, pyrimidy1-H), 6.14 (t, *J* = 7.5 Hz, 1H, CH), 5.37 (s, 2H, CH_2), 4.05 (s, 3H, CH_3), 3.89 (d, *J* = 7.5 Hz, 2H, CH_2), 3.88 (s, 3H, CH_3). ^{13}C NMR (125 MHz, $CDCl_3$) δ 172.36, 169.21, 163.21, 156.25 (q, *J* = 36.3 Hz), 149.20, 133.45, 130.13, 129.62, 128.93, 128.76, 128.56, 125.24, 123.93, 123.46, 120.18 (q, *J* = 273.8 Hz), 101.08 (q, *J* = 2.5 Hz), 67.74, 63.90, 53.03, 29.70. ^{19}F NMR (470 MHz, $CDCl_3$) δ -70.53.

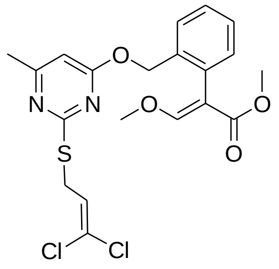

Data for
(*E*)-methyl 2-(2-((2-(3,3-dichloroallyloxy)-6-methylpyrimidin-4-yloxy)methyl)phenyl)-3-methoxyacrylate (**4r**). Yellow oil, yield, 83%. ^{1}H NMR (500 MHz, $CDCl_3$) δ 7.59 (s, 1H, CH), 7.48-7.49 (m, 1H, Ar-H), 7.35-7.38 (m, 2H, Ar-H), 7.20-7.22 (m, 1H, Ar-H), 6.29 (s, 1H, pyrimidy1-H), 6.11 (t, *J* = 8.0 Hz, 1H, CH), 5.32 (s, 2H, CH_2), 3.85 (d, *J* = 8.0 Hz, 2H, CH_2), 3.83 (s, 3H, CH_3), 3.71 (s, 3H, CH_3), 2.39 (s, 3H, CH_3). ^{13}C NMR (125 MHz, $CDCl_3$) δ 169.48, 169.40, 167.98, 169.77, 160.03, 135.11, 131.84, 131.21, 128.21, 128.04, 127.98, 126.34, 122.86, 110.08, 102.67, 66.47, 62.00, 51.67, 29.44, 23.74.

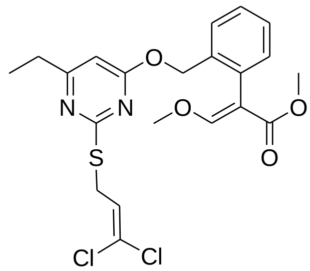

Data for
(*E*)-methyl 2-(2-((2-(3,3-dichloroallyloxy)-6-ethylpyrimidin-4-yloxy)methyl)phenyl)-3-methoxyacrylate (**4s**). Yellow oil, yield, 80%. ^{1}H NMR (500 MHz, $CDCl_3$) δ 7.60 (s, 1H, CH), 7.48-7.50 (m, 1H, Ar-H), 7.36-7.38 (m, 2H, Ar-H), 7.20-7.22 (m, 1H, Ar-H), 6.29 (s, 1H, pyrimidy1-H), 6.13 (t, *J* = 7.5 Hz, 1H, CH), 5.32 (s, 2H, CH_2), 3.86 (d, *J* = 7.5 Hz, 2H, CH_2), 3.84 (s, 3H, CH_3), 3.71 (s, 3H, CH_3), 2.67 (q, *J* =7 .5 Hz, 2H, CH_2), 1.27 (t, *J*=7.5 Hz, 3H, CH_3). ^{13}C NMR (125 MHz, $CDCl_3$) δ 172.98, 169.37, 169.33, 160.03, 135.17, 131.90, 131.19, 128.31, 128.03, 127.98, 126.48, 122.76, 110.10, 101.41, 66.44, 62.00, 51.66, 30.51, 29.46, 12.56.

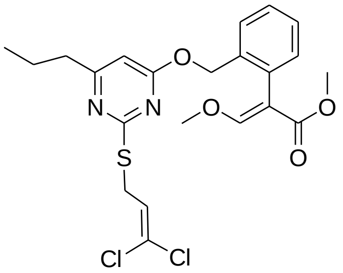

Data for
(*E*)-methyl 2-(2-((2-(3,3-dichloroallyloxy)-6-propylpyrimidin-4-yloxy)methyl)phenyl)-3-methoxyacrylate (**4t**). Yellow oil, yield, 76%. ^{1}H NMR (500 MHz, $CDCl_3$) δ 7.59 (s, 1H, CH), 7.49-7.51 (m, 1H, Ar-H), 7.36-7.38 (m, 2H, Ar-H), 7.20-7.22 (m, 1H, Ar-H), 6.28 (s, 1H, pyrimidy1-H), 6.13 (t, *J* = 7.5 Hz, 1H, CH), 5.32 (s, 2H, CH_2), 3.86 (d, *J* = 7.5 Hz, 2H, CH_2), 3.84 (s, 3H, CH_3), 3.71 (s, 3H, CH_3), 2.60 (t, *J* = 7.5 Hz, 2H, CH_2), 1.69-1.76 (m, 2H, CH_2), 0.98 (t, *J* = 7.5 Hz, 3H, CH_3). ^{13}C NMR (125 MHz, $CDCl_3$) δ 171.62, 169.38, 169.22, 167.79, 160.04, 135.10, 131.90, 131.20, 128.32, 128.05, 128.00, 126.41, 122.82, 110.09, 102.19, 66.50, 62.00, 51.68, 39.30, 29.47, 21.76, 13.75.

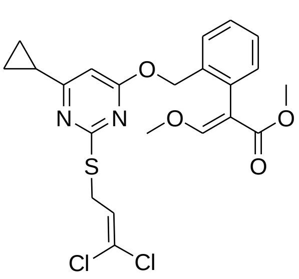

Data for
(*E*)-methyl 2-(2-((6-cyclopropyl-2-(3,3-dichloroallyloxy)pyrimidin-4-yloxy)methyl)phenyl)-3-methoxyacrylate (**4u**). Colorless oil, yield, 81%. ^{1}H NMR (500 MHz, $CDCl_3$) δ 7.59 (s, 1H, CH) , 7.48-7.50 (m, 1H, Ar-H), 7.35-7.37 (m, 2H, Ar-H), 7.19-7.21 (m, 1H, Ar-H), 6.28 (s, 1H, pyrimidy1-H), 6.09 (t, *J* = 7.5 Hz, 1H), 5.30 (s, 2H, CH_2), 3.84 (s, 3H, CH_3), 3.80 (d, *J* = 7.0 Hz, 2H, CH_2), 3.71 (s, 3H, CH_3), 1.86-1.90 (m, 1H, cyclopropyl-CH), 1.09-1.12 (m, 2H, cyclopropyl-CH_2), 1.00-1.04 (m, 2H, cyclopropyl-CH_2). ^{13}C NMR (125 MHz, $CDCl_3$) δ 172.58, 169.29, 168.76, 167.82, 160.03, 135.23, 131.16, 128.36, 128.02, 127.96, 126.56, 122.71, 110.09, 100.06, 66.42, 62.01, 51.68, 29.71, 29.38, 16.73, 10.34. HRMS m/z 503.0567 $[M + Na]^+$(calcd $[M + Na]^+$ 503.0570).

2. Spectrogram of title compounds 4a-4u

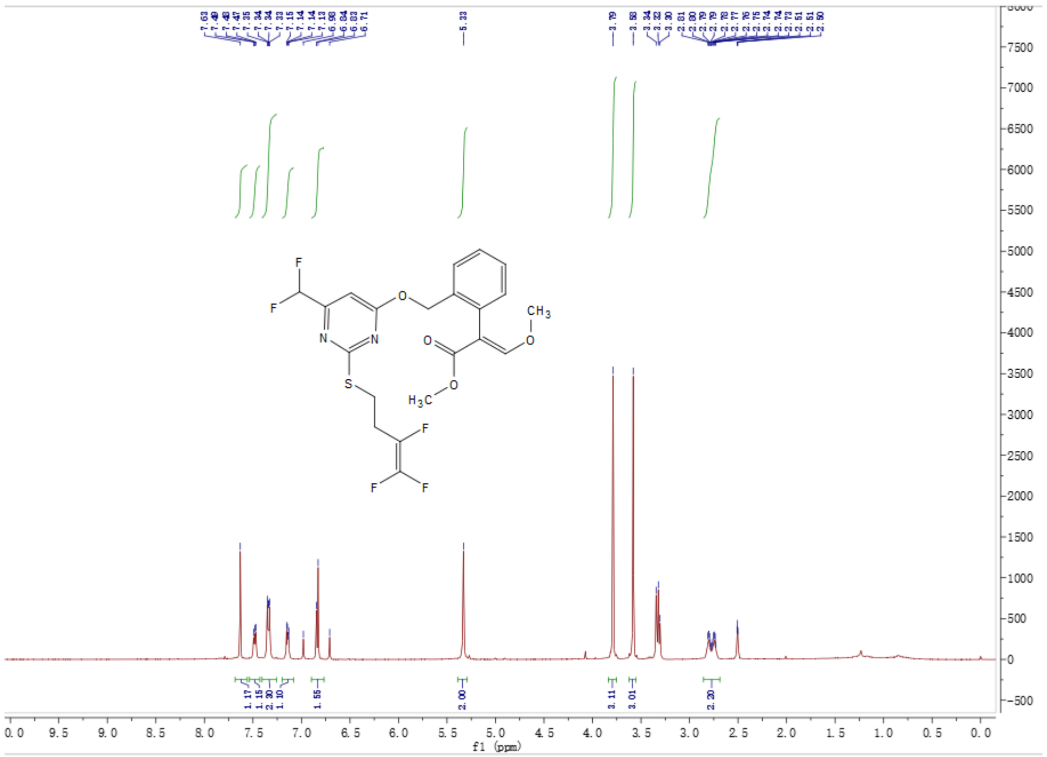

Fig. S1 ^{1}H NMR of compound **4a**

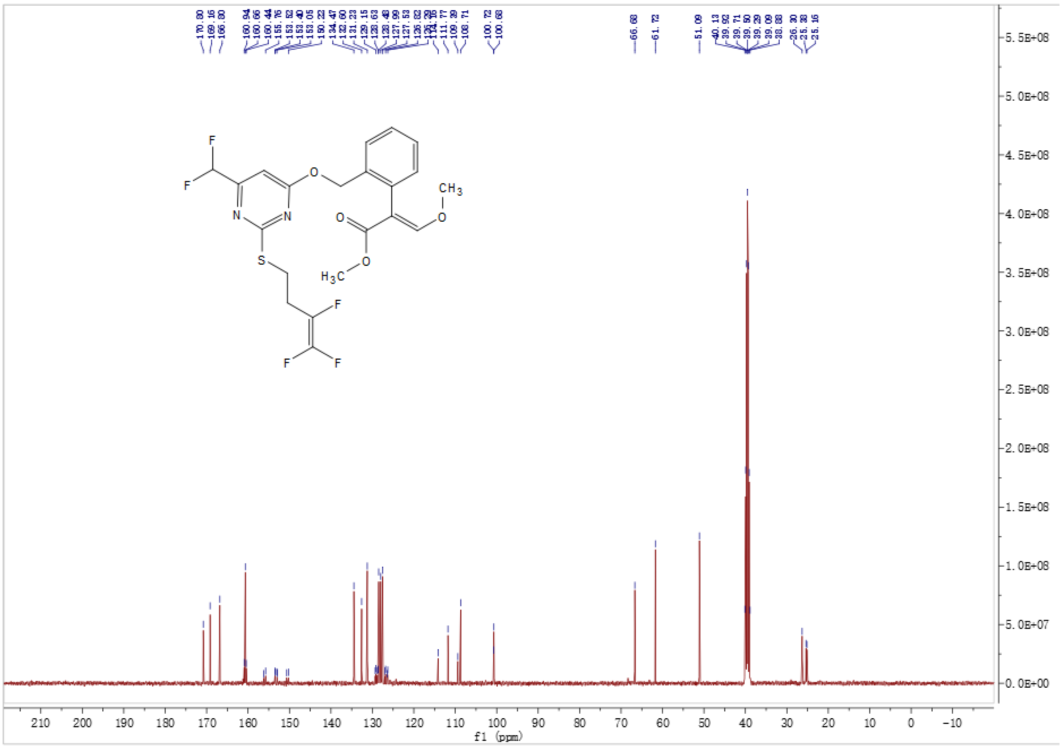

Fig. S2 ^{13}C NMR of compound **4a**

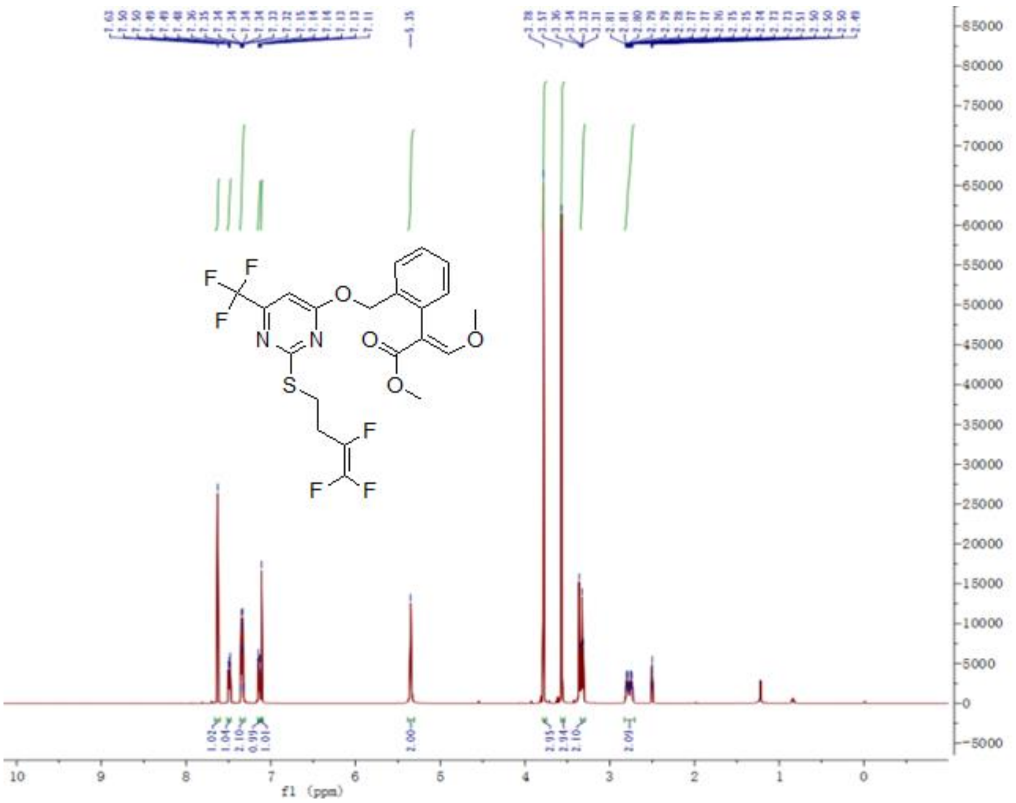

Fig. S3 ^{1}H NMR of compound **4b**

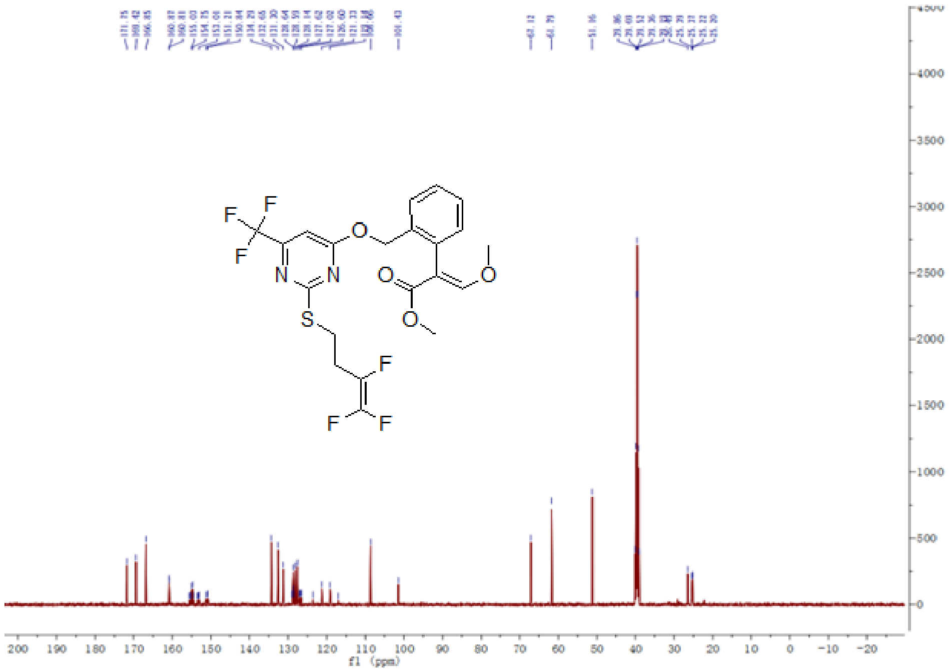

Fig. S4 ^{13}C NMR of compound **4b**

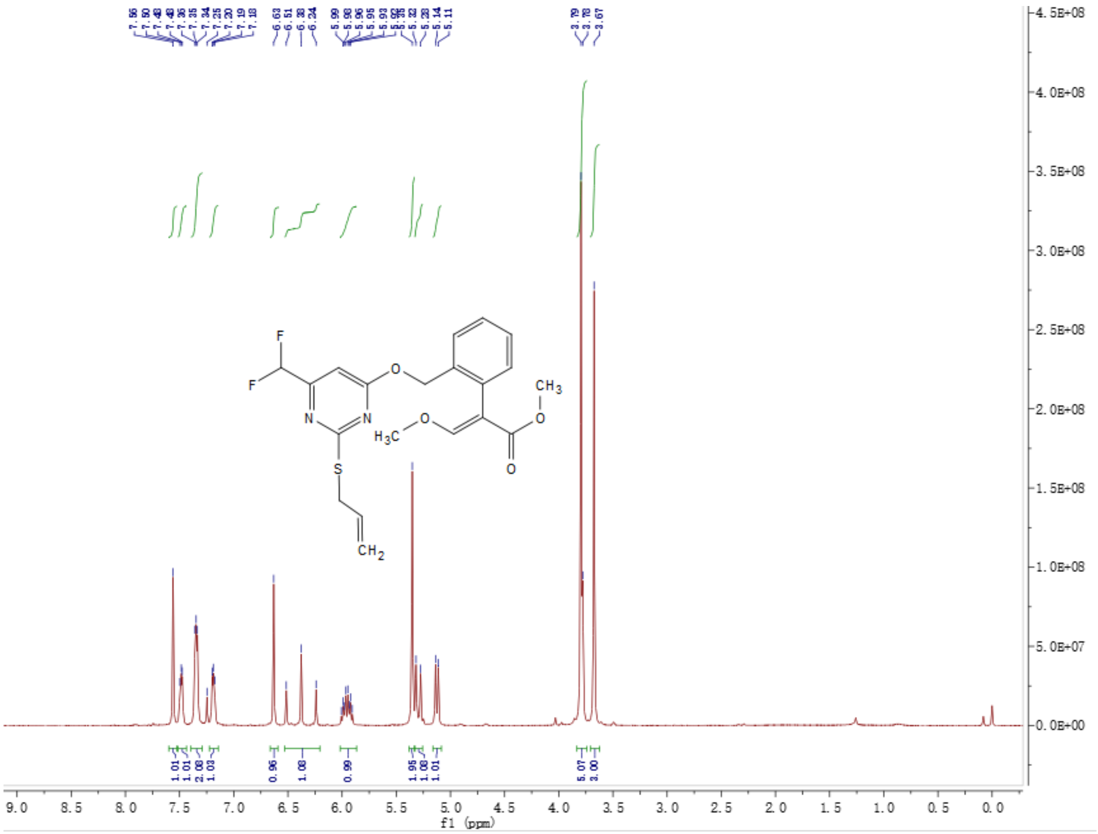

Fig. S5 ^{1}H NMR of compound **4c**

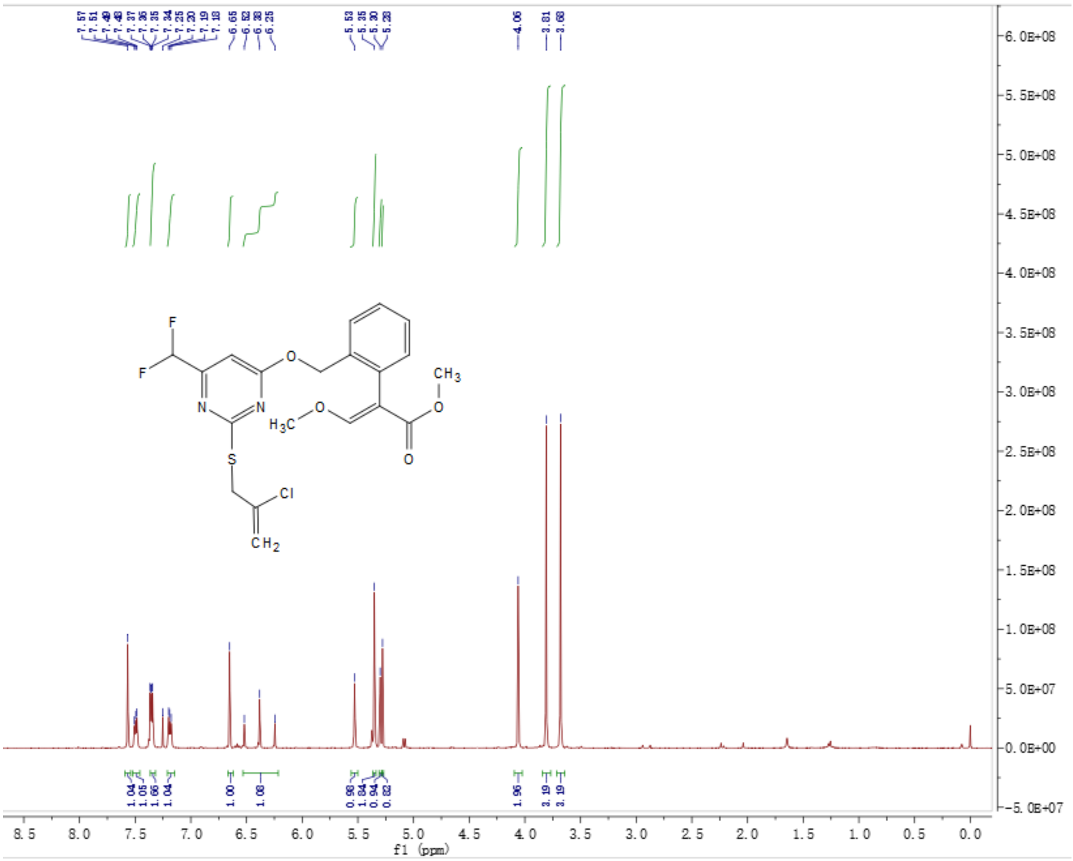

Fig. S6 ^{1}H NMR of compound **4d**

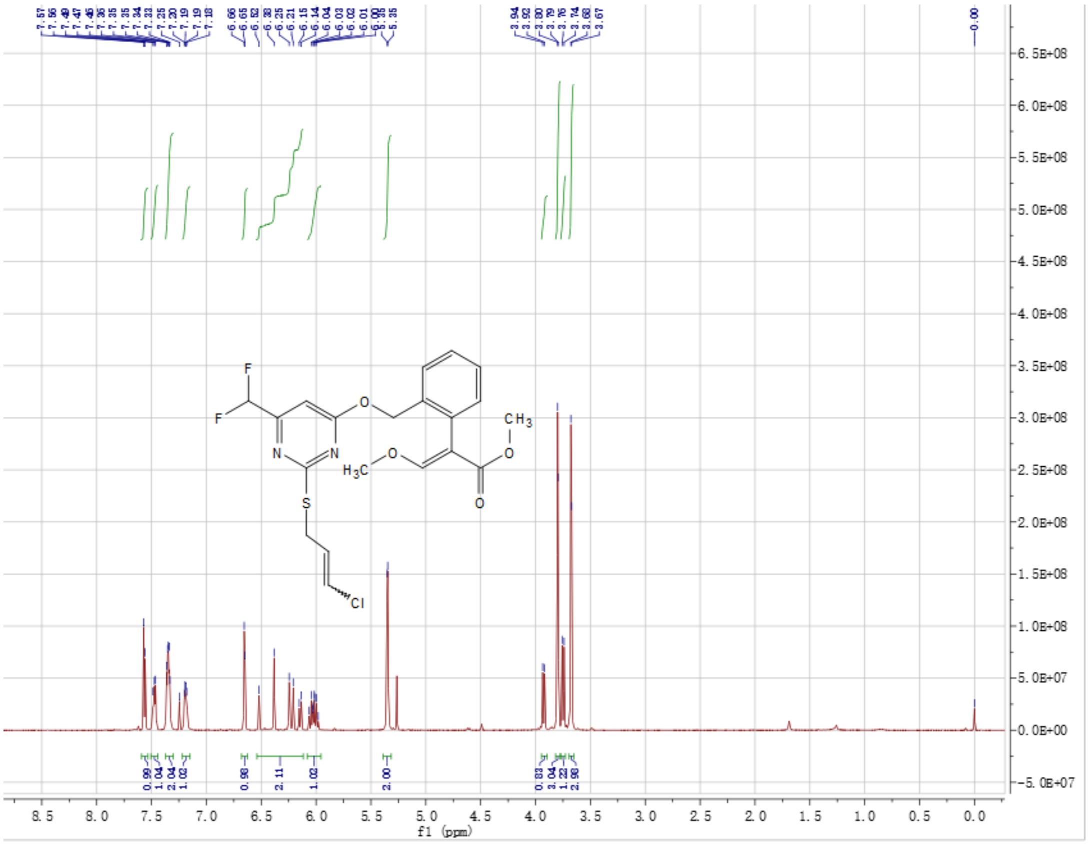

Fig. S7 ¹H NMR of compound **4e**

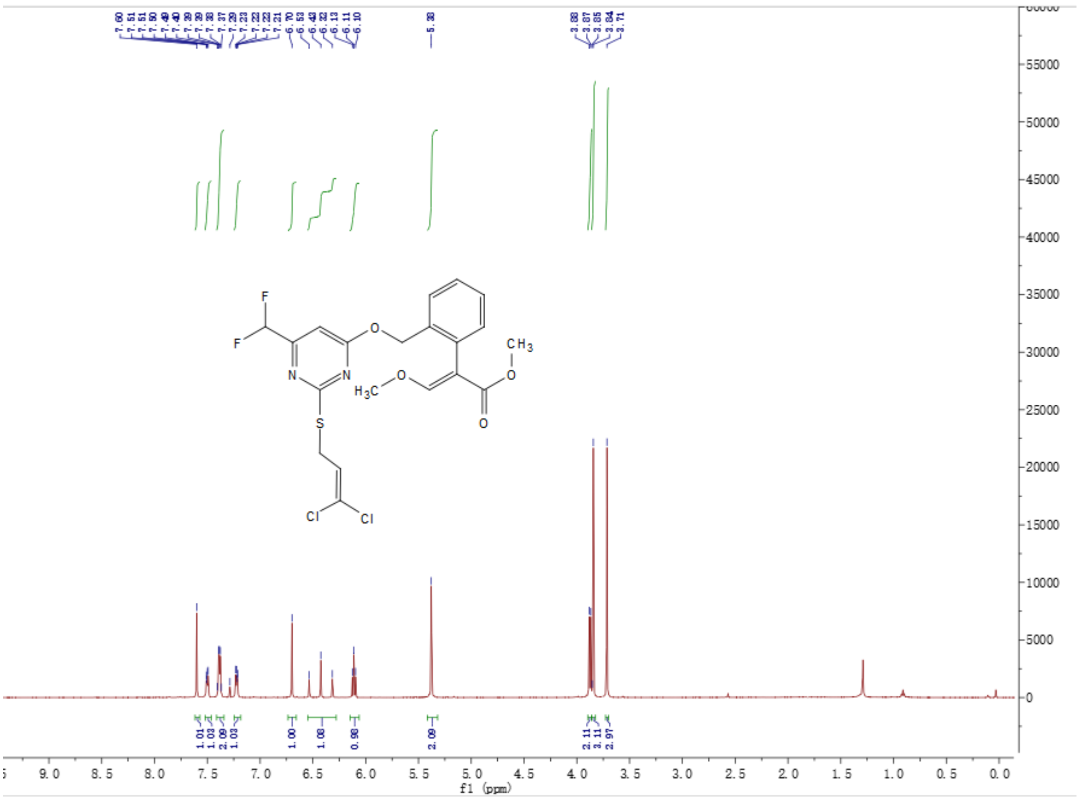

Fig. S8 ¹H NMR of compound **4f**

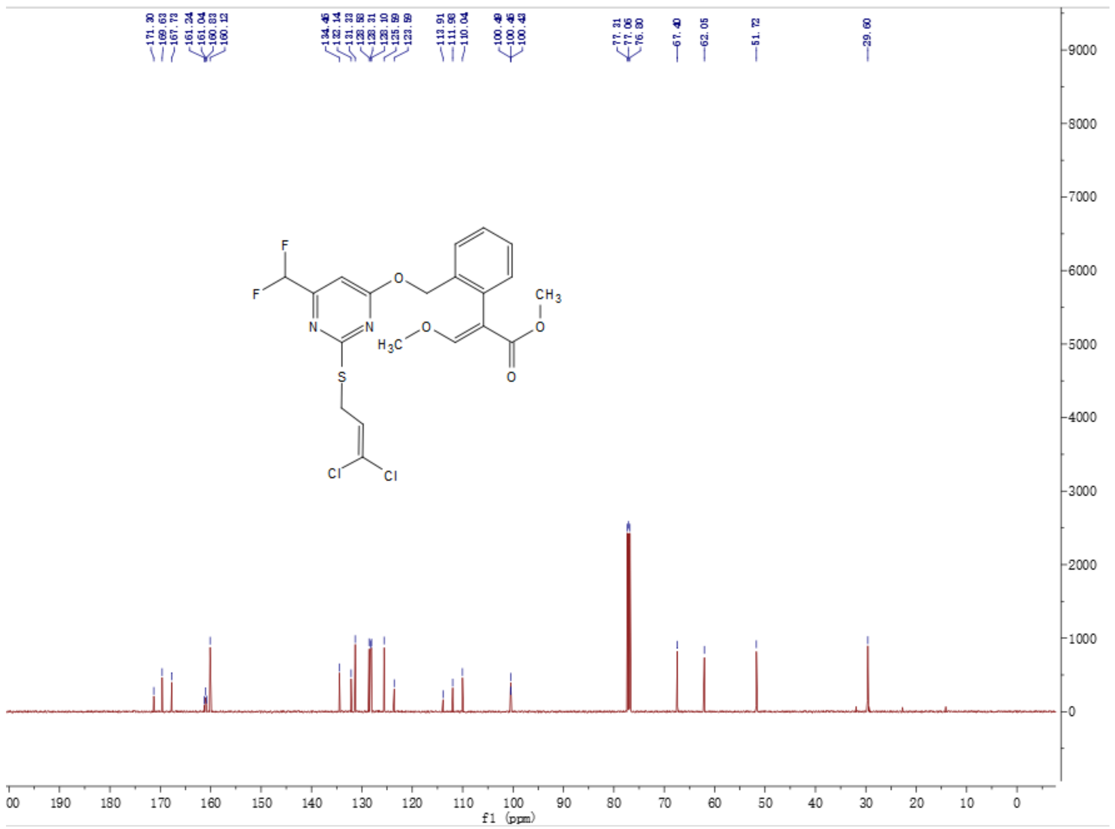

Fig. S9 ^{13}C NMR of compound **4f**

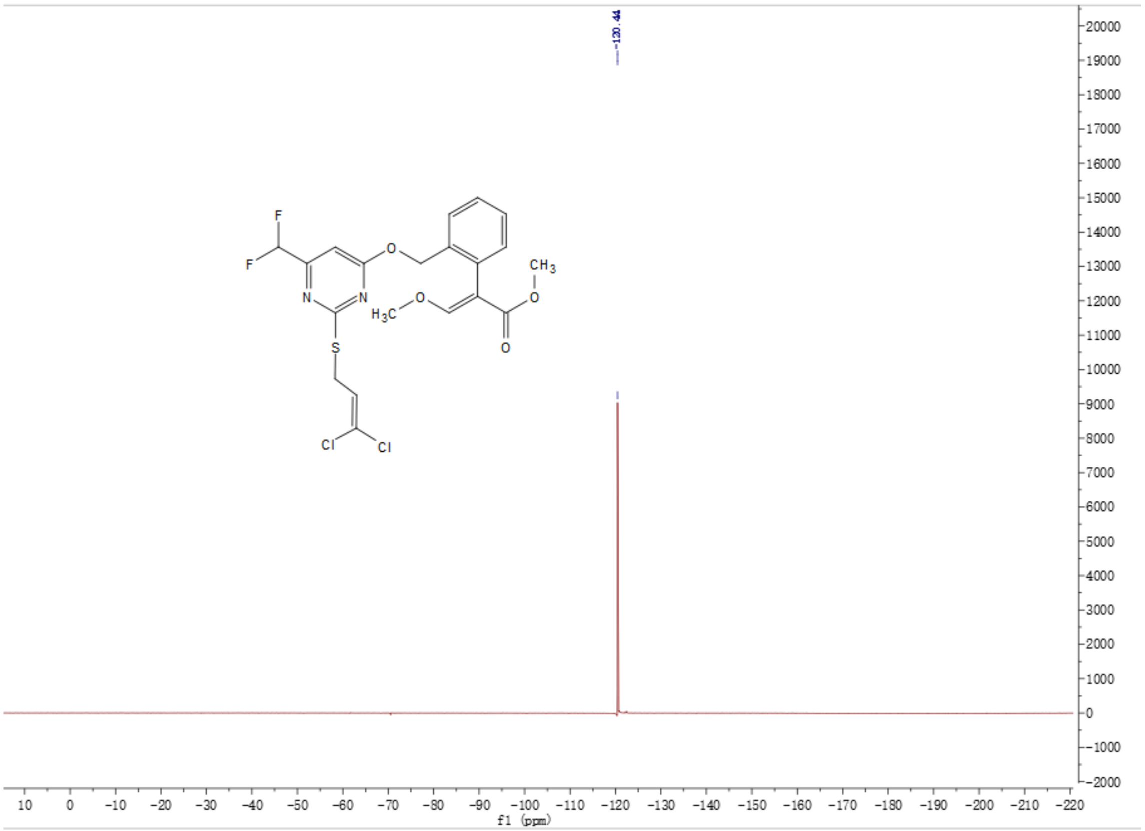

Fig. S10 ^{19}F NMR of compound **4f**

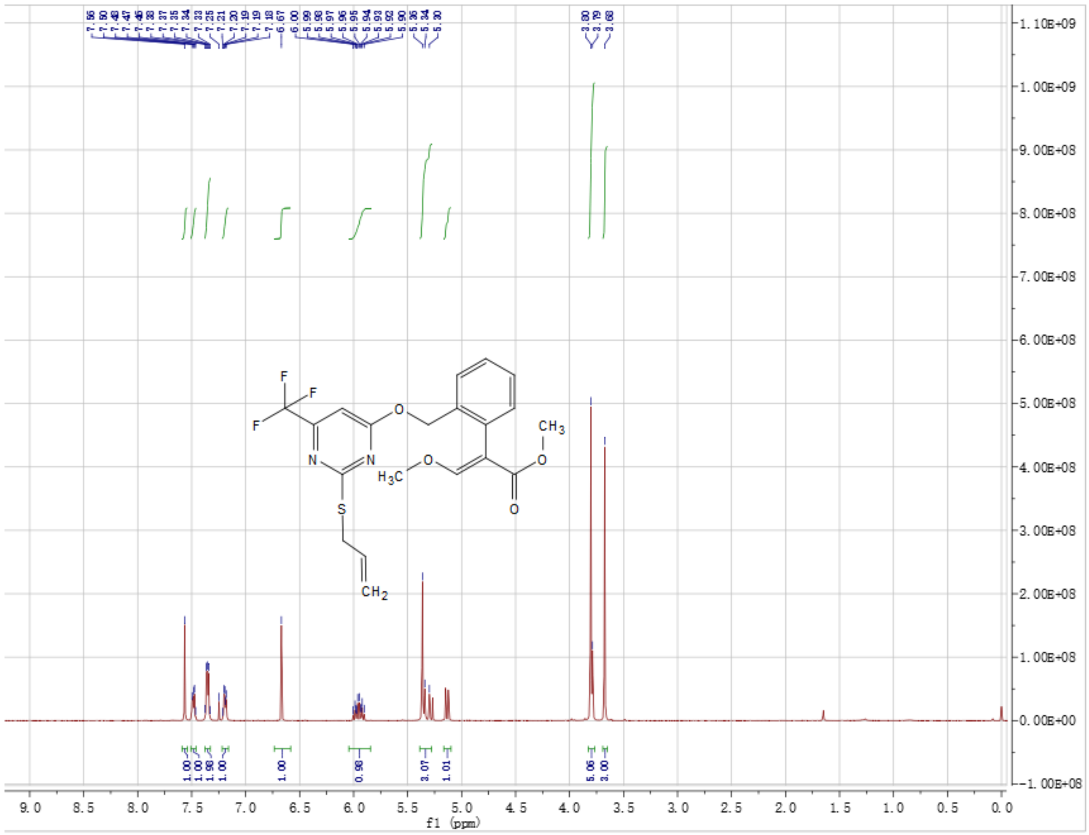

Fig. S11 ^{1}H NMR of compound **4g**

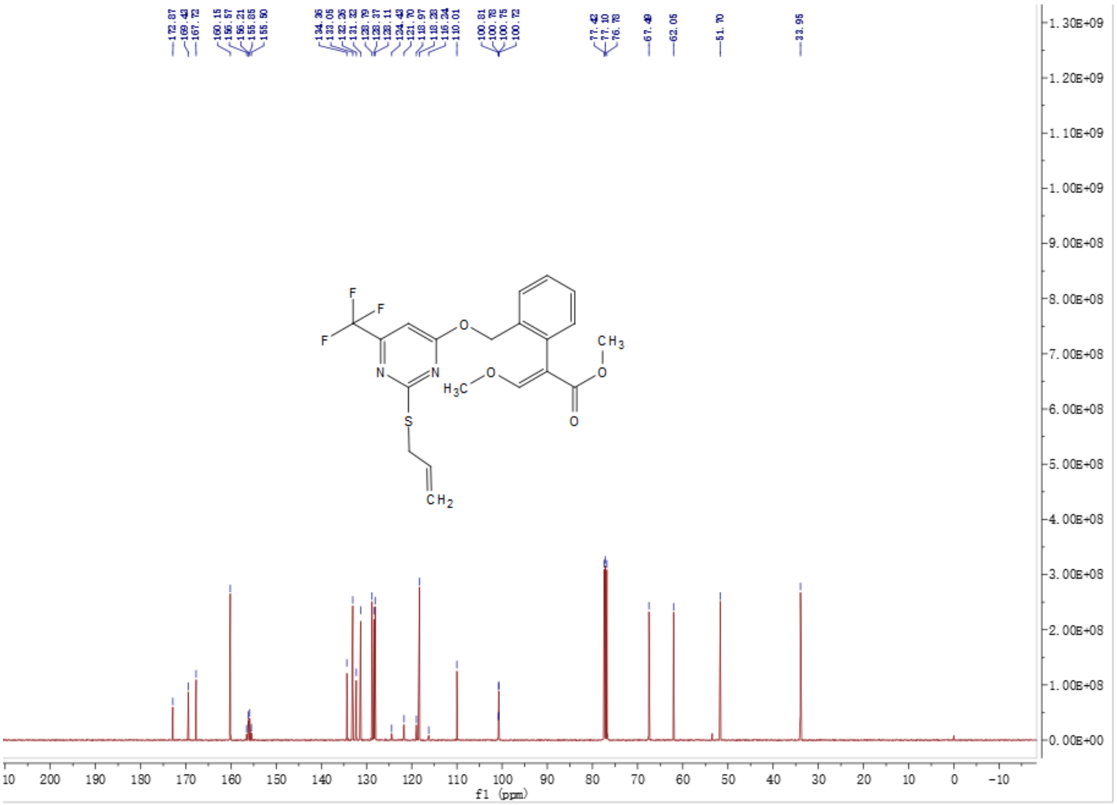

Fig. S12 ^{13}C NMR of compound **4g**

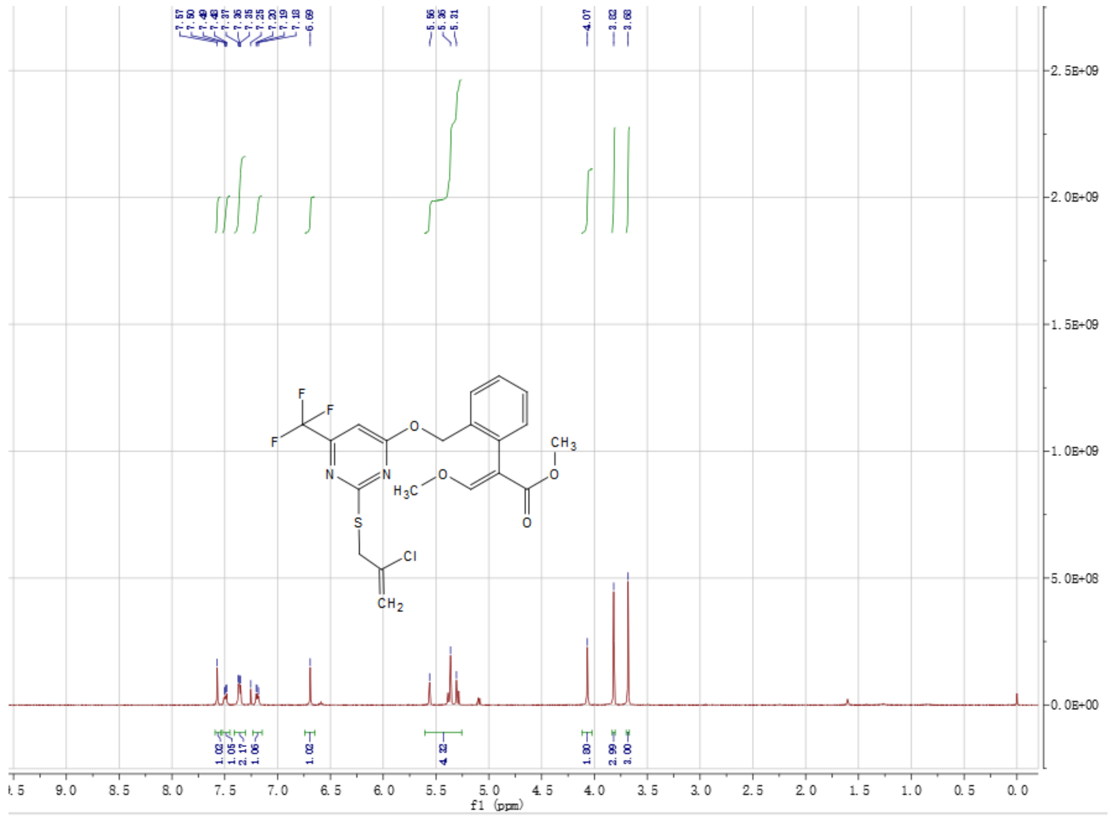

Fig. S13 ^1H NMR of compound **4h**

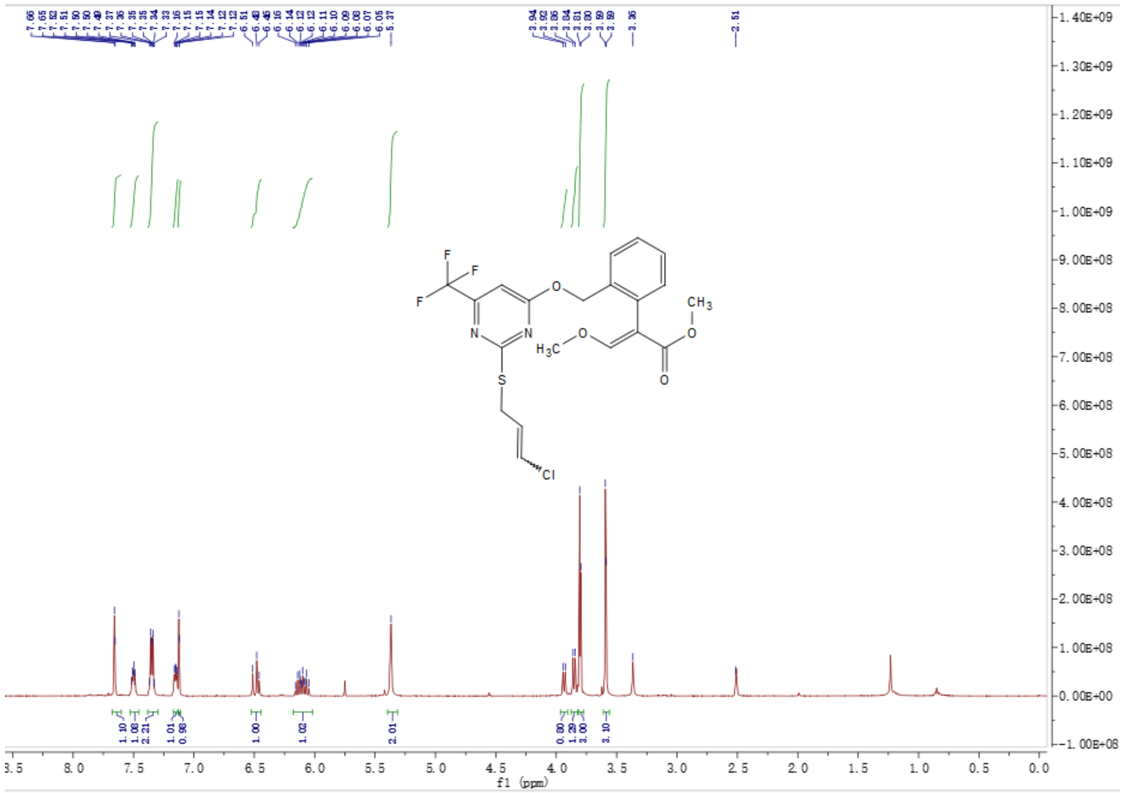

Fig. S14 ^1H NMR of compound **4i**

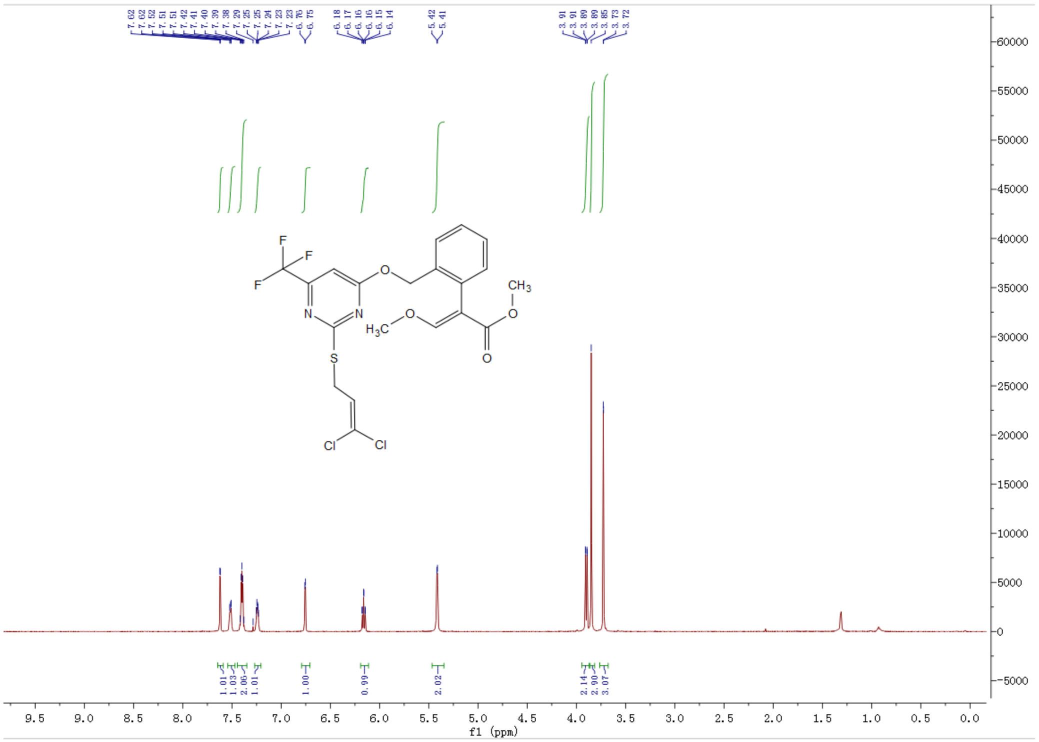

Fig. S15 ¹H NMR of compound **4j**

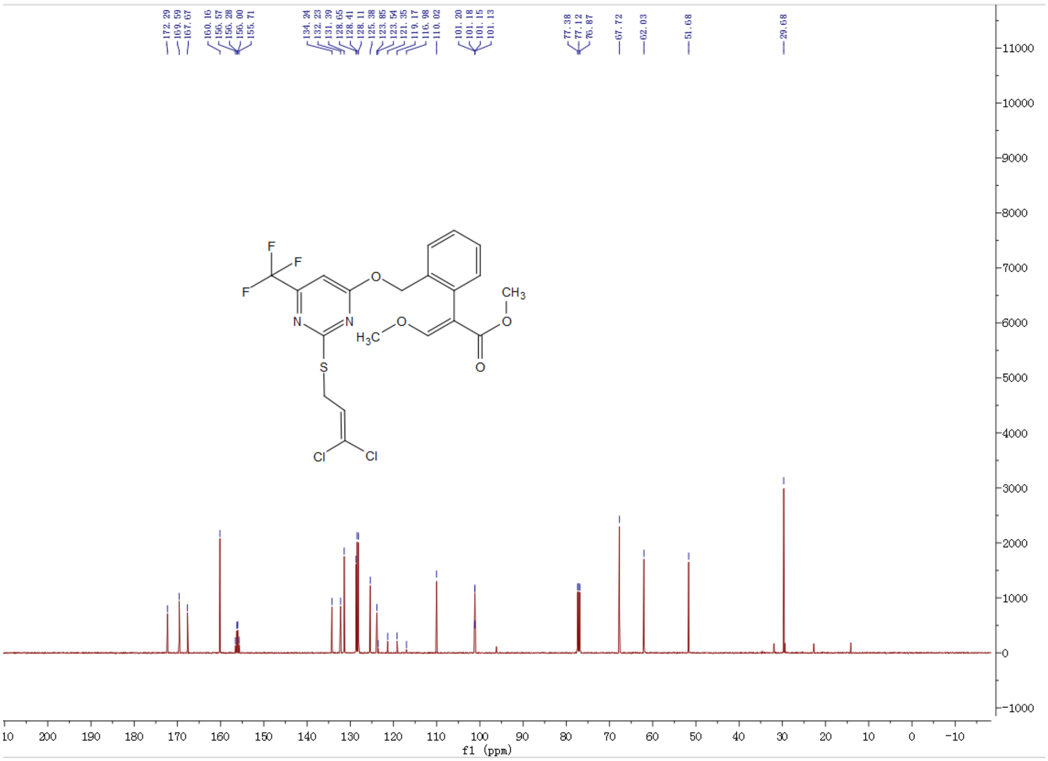

Fig. S16 ¹³C NMR of compound **4j**

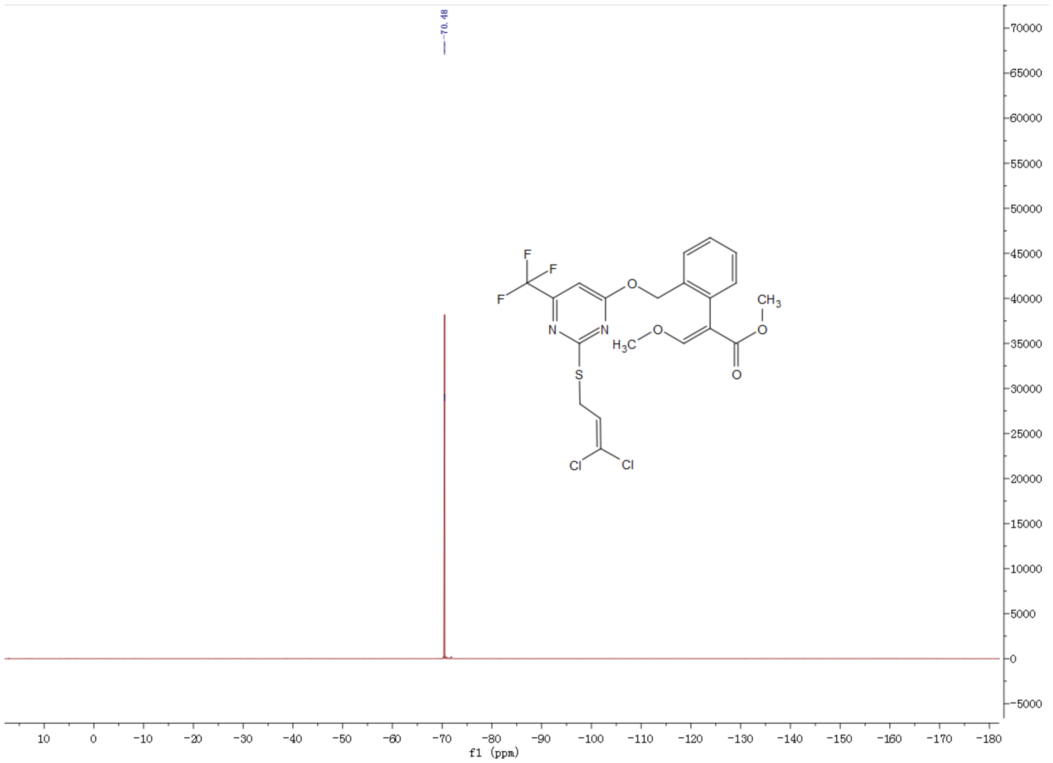

Fig. S17 ^{19}F NMR of compound **4j**

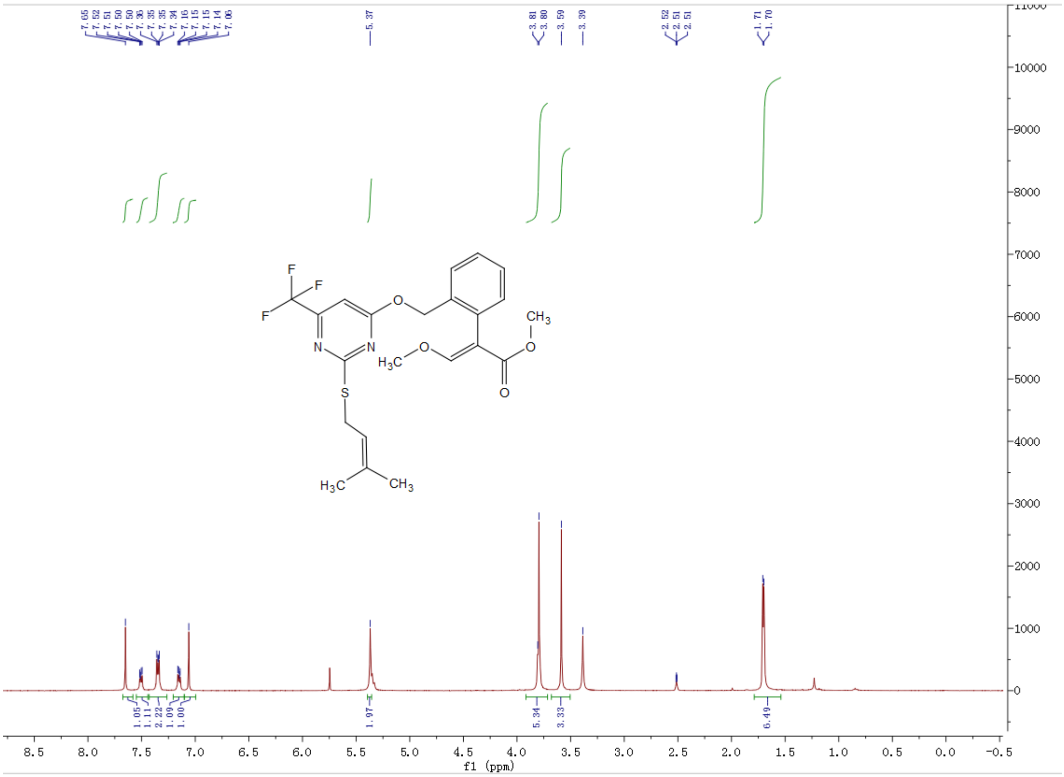

Fig. S18 ^{1}H NMR of compound **4k**

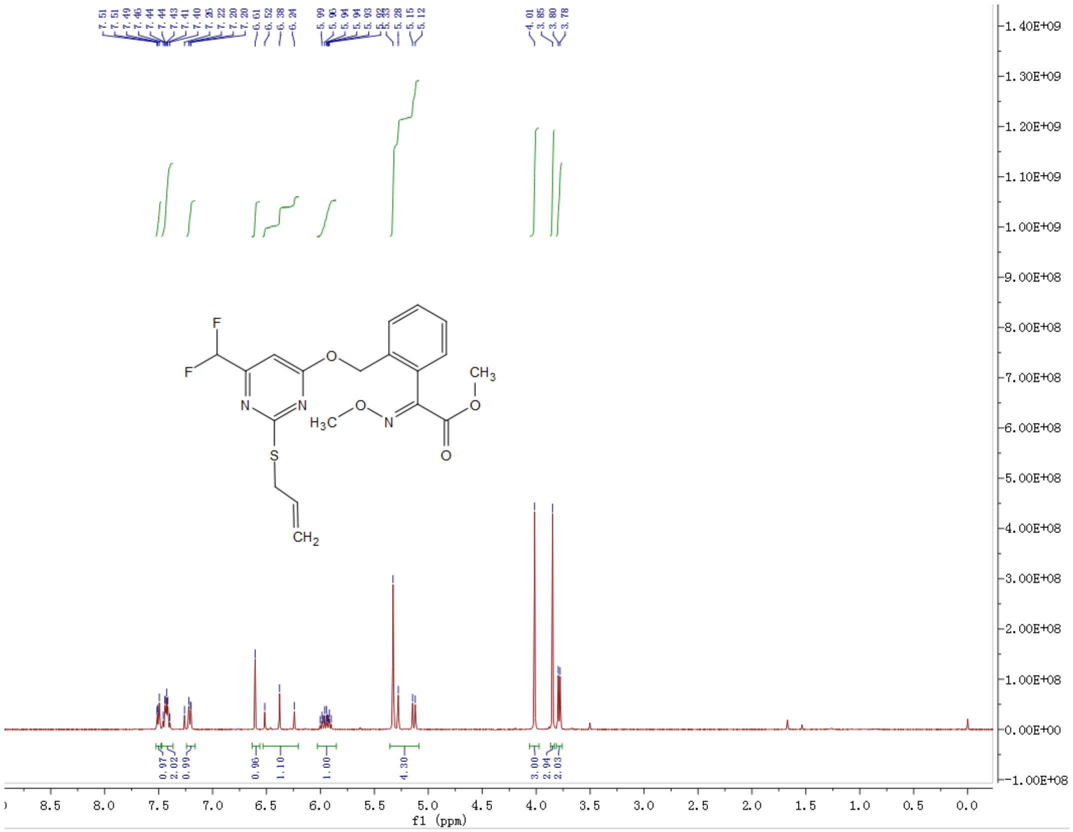

Fig. S19 ^{1}H NMR of compound **4l**

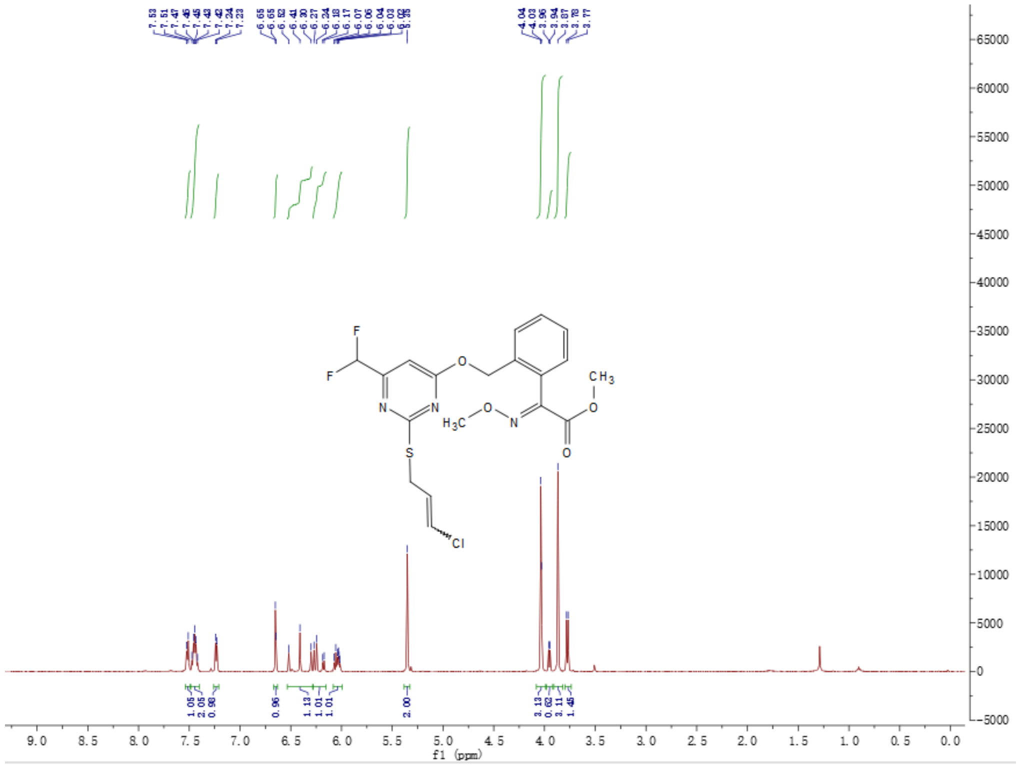

Fig. S20 ^{1}H NMR of compound **4m**

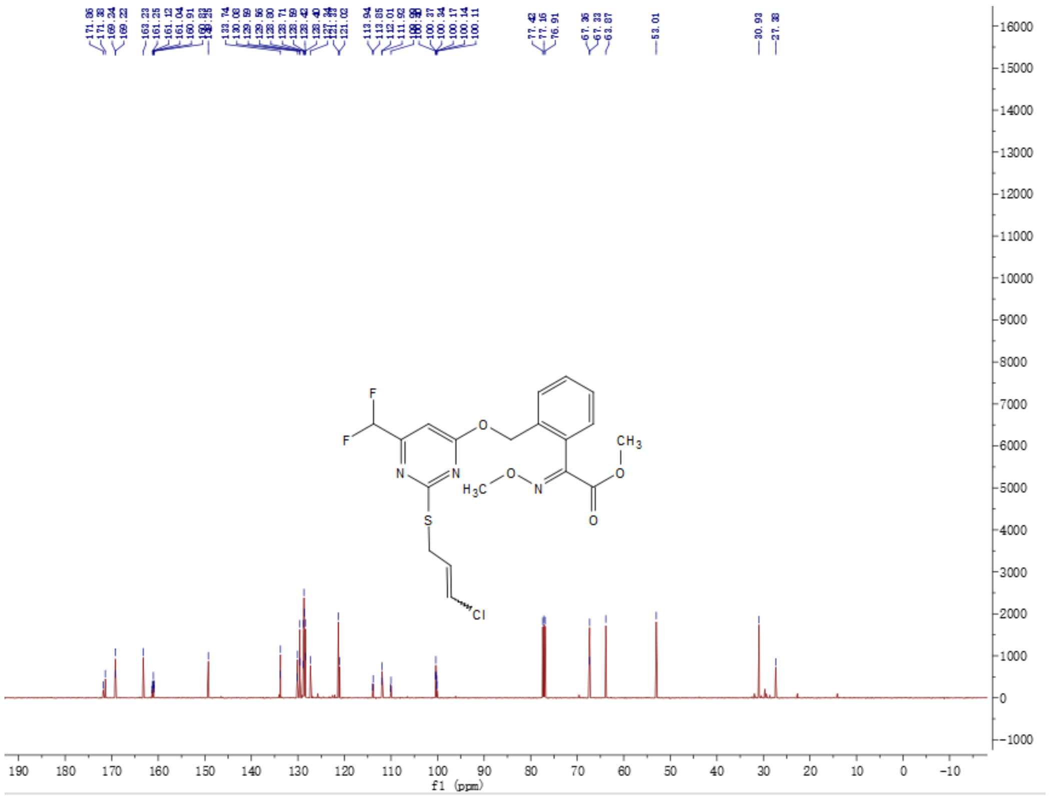

Fig. S21 ^{13}C NMR of compound **4m**

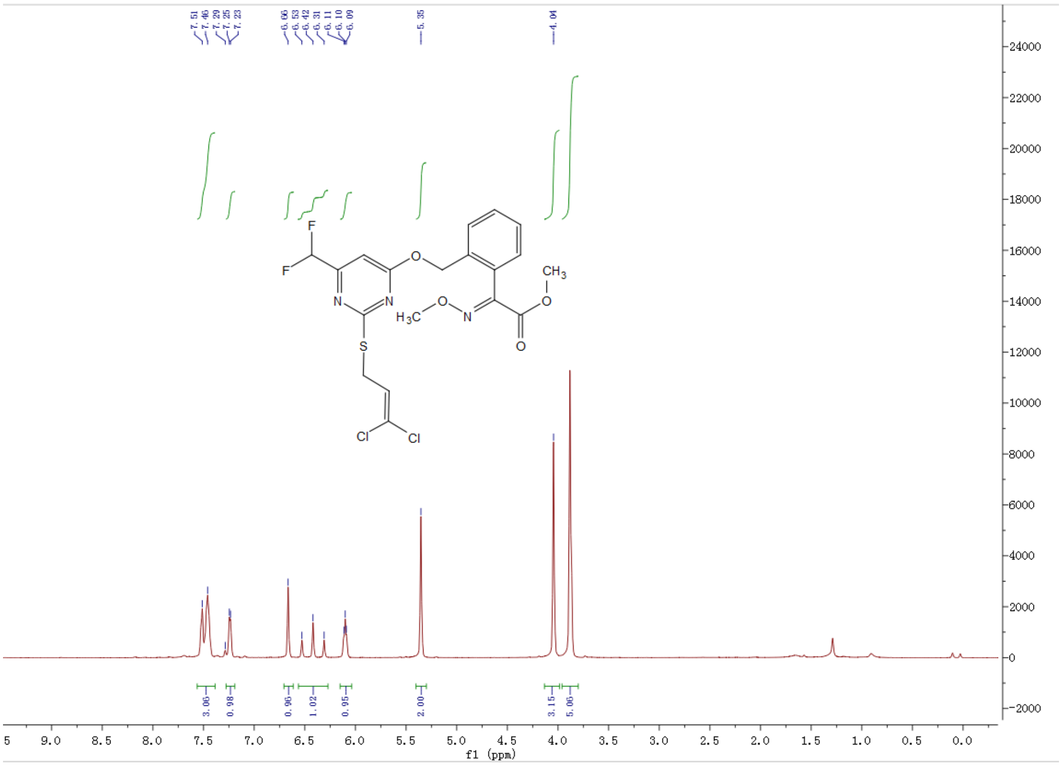

Fig. S22 ^{1}H NMR of compound **4n**

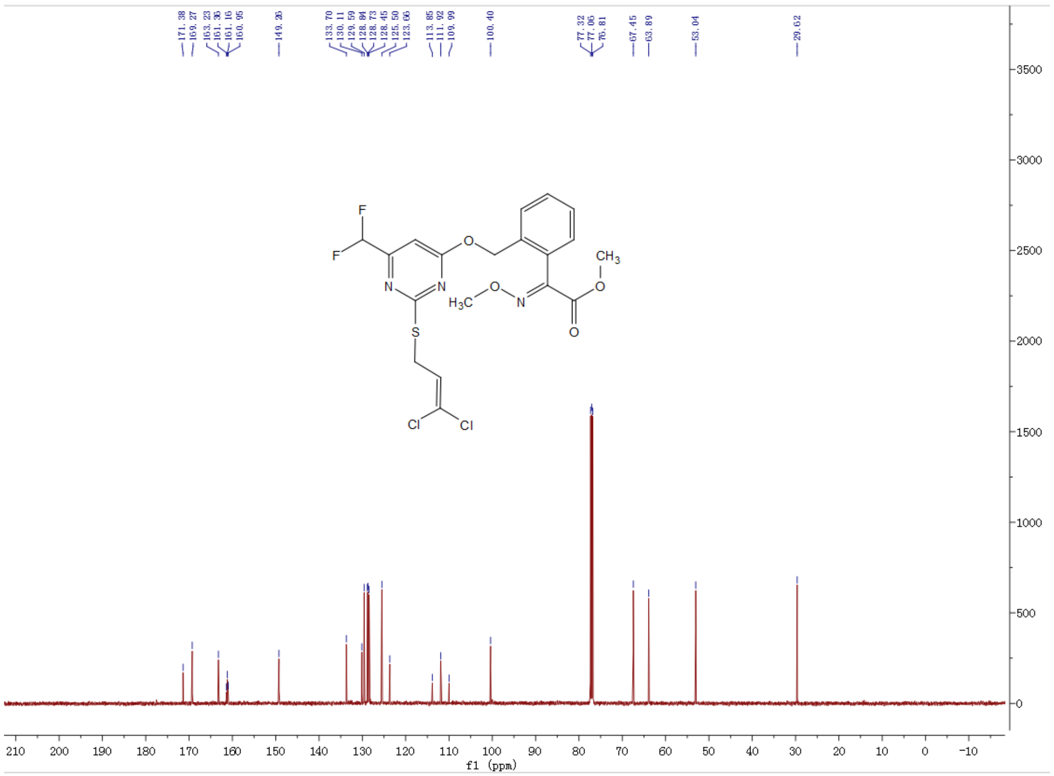

Fig. S23 ^{13}C NMR of compound **4n**

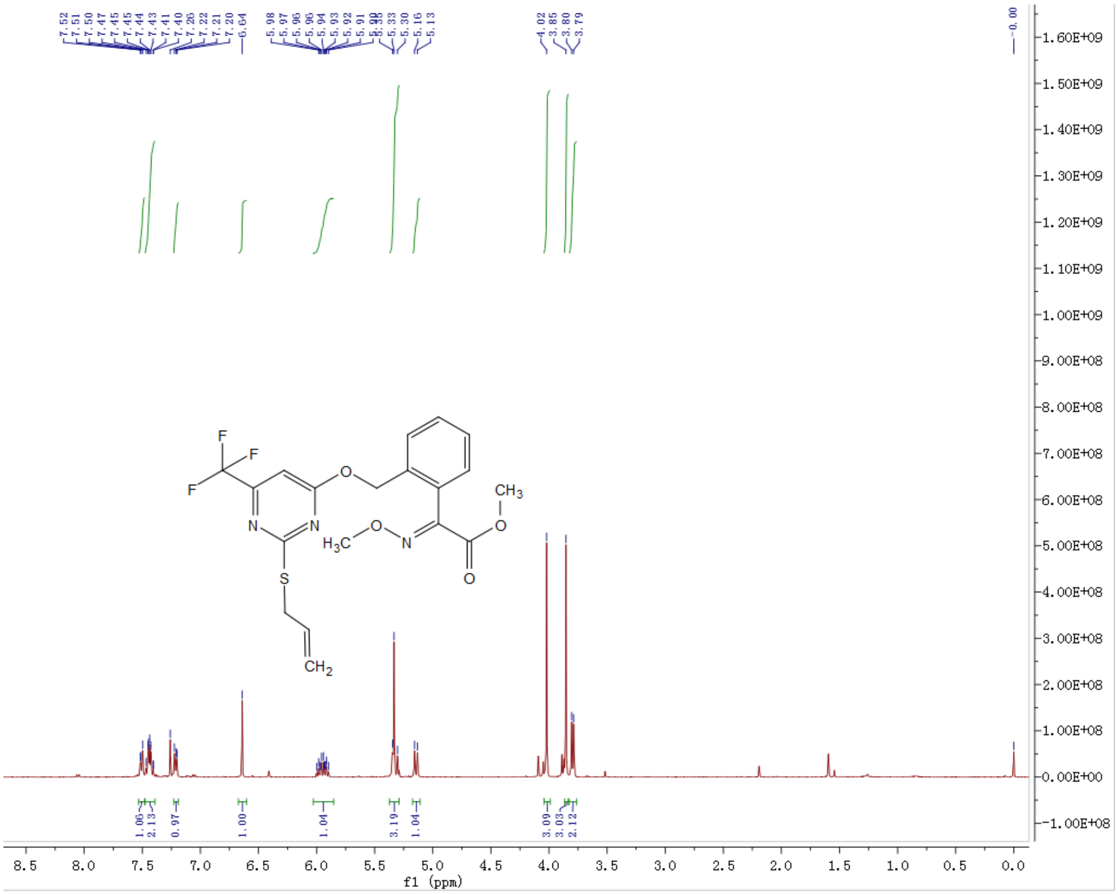

Fig. S24 ^{1}H NMR of compound **4o**

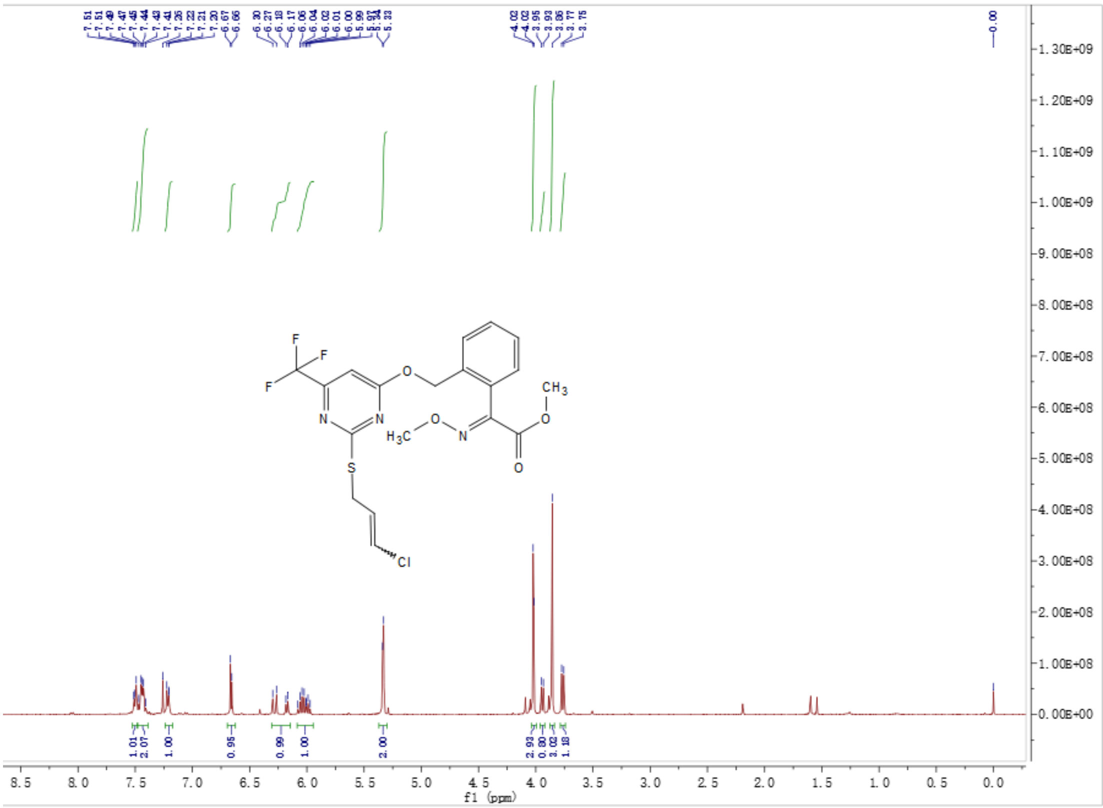

Fig. S25 ^{1}H NMR of compound **4p**

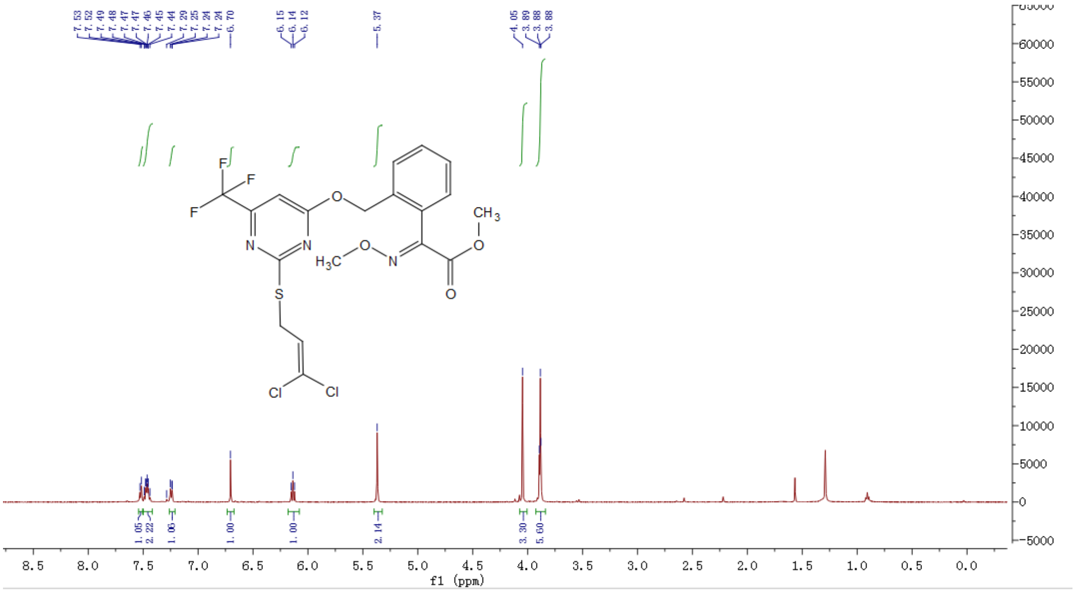

Fig. S26 ^{1}H NMR of compound **4q**

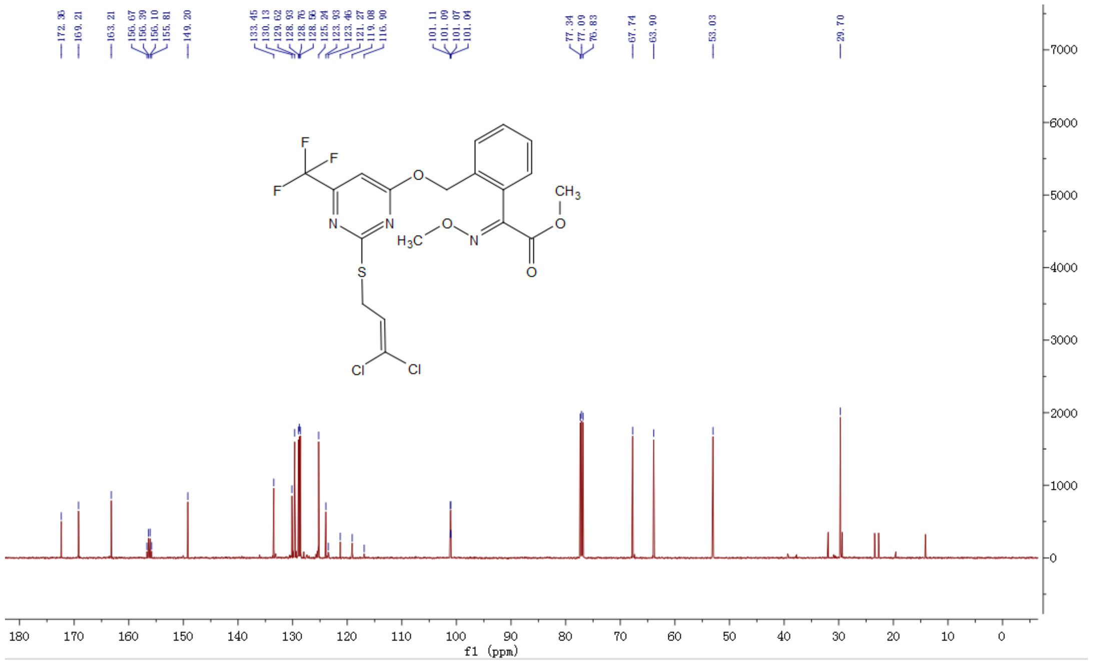

Fig. S27 ^{13}C NMR of compound **4q**

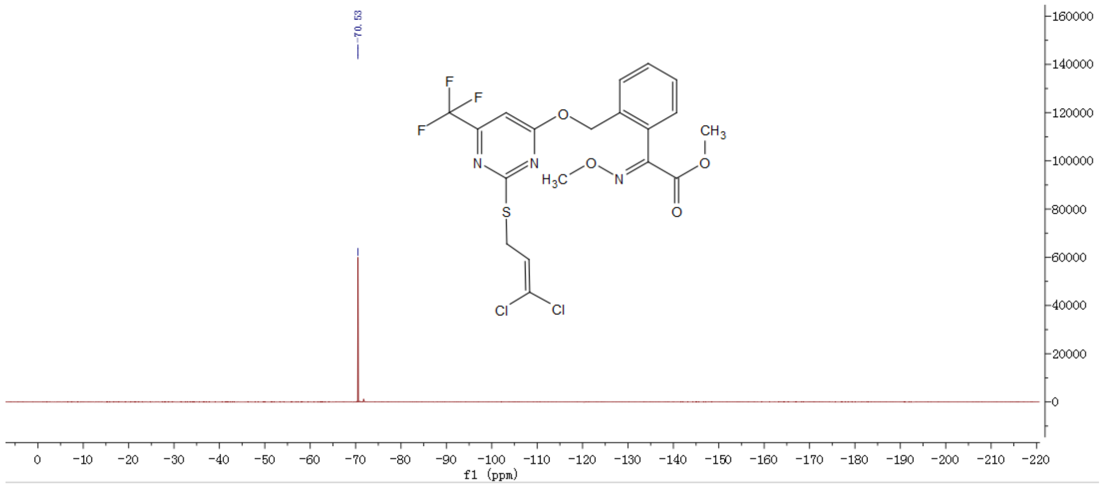

Fig. S28 ^{19}F NMR of compound **4q**

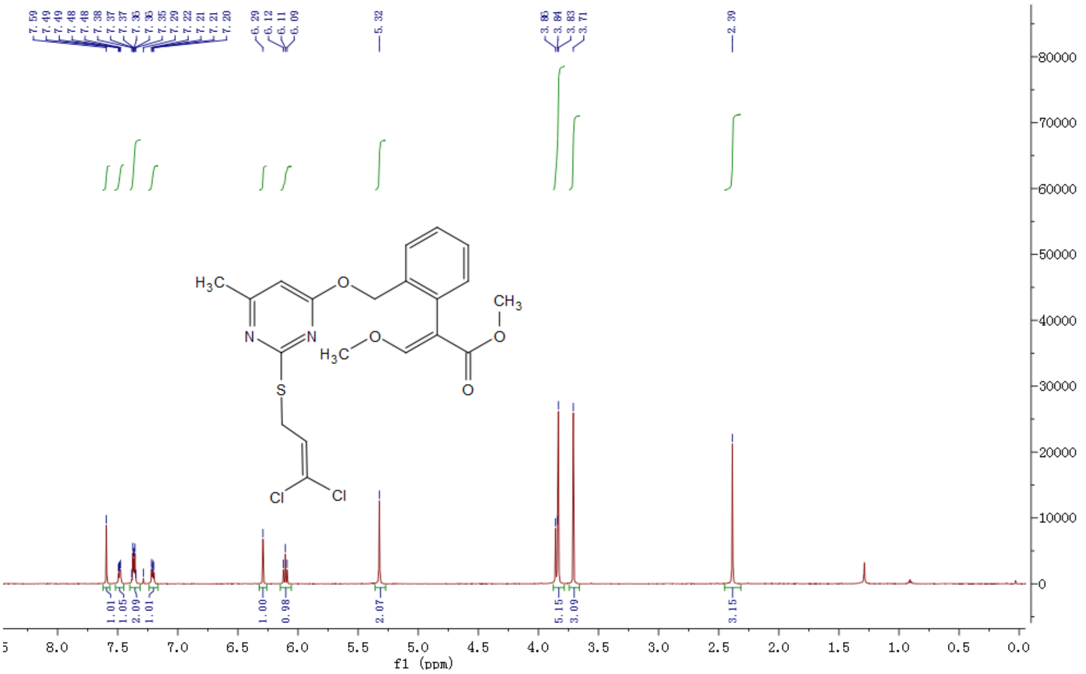

Fig. S29 ^{1}H NMR of compound **4r**

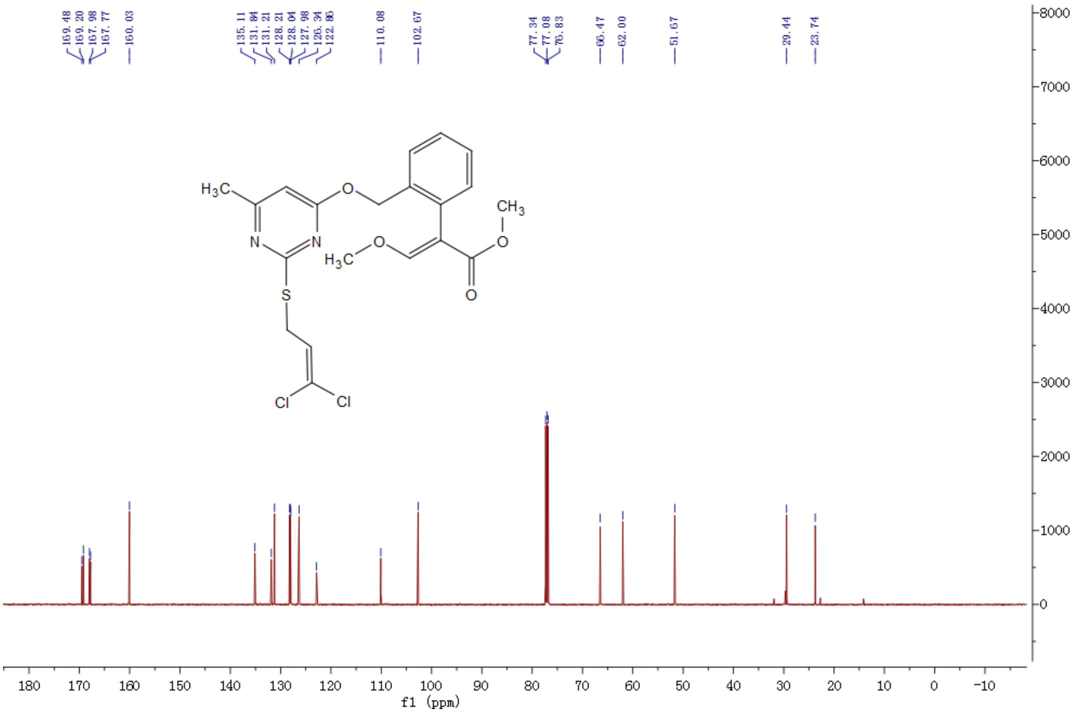

Fig. S30 ^{13}C NMR of compound **4r**

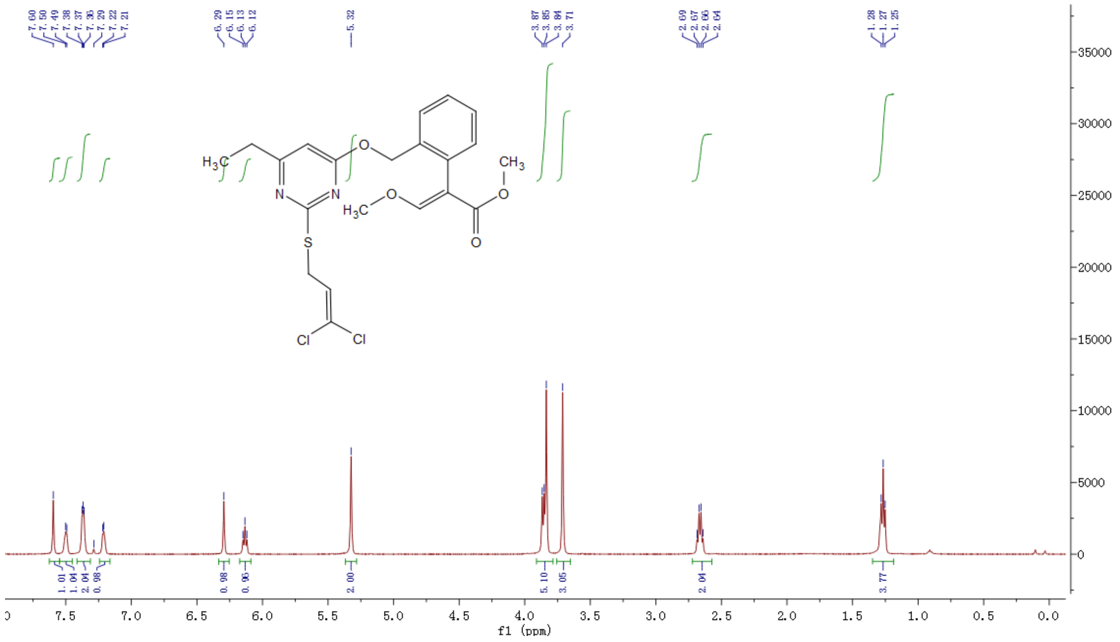

Fig. S31 ^{1}H NMR of compound **4s**

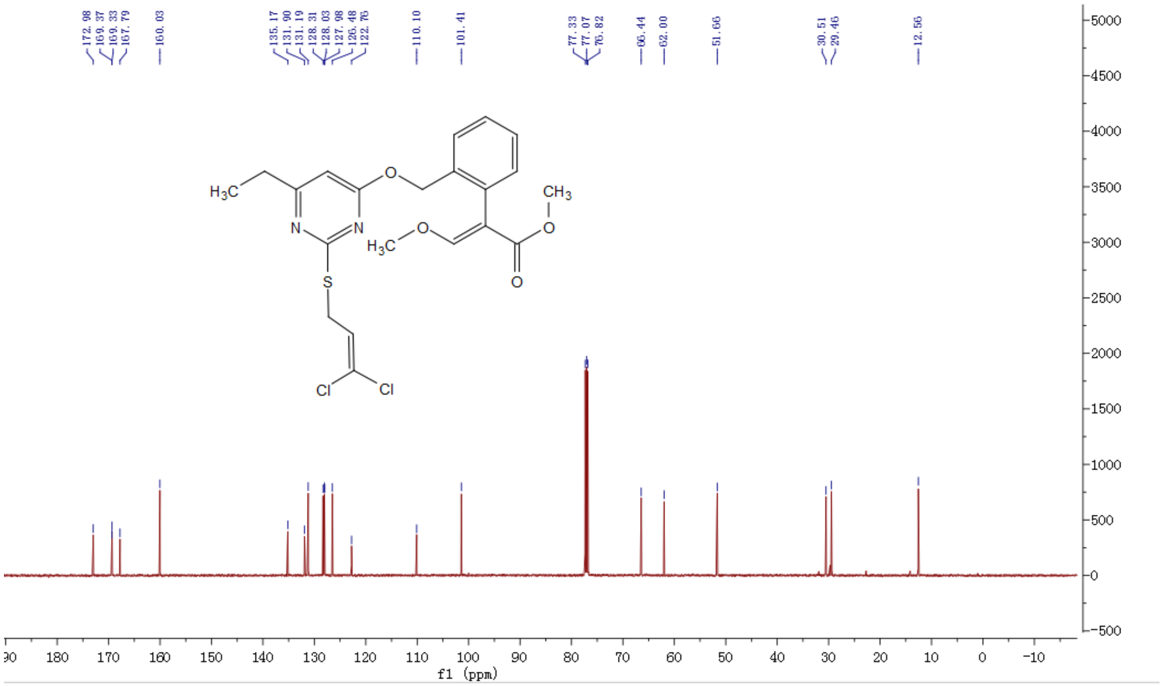

Fig. S32 ^{13}C NMR of compound **4s**

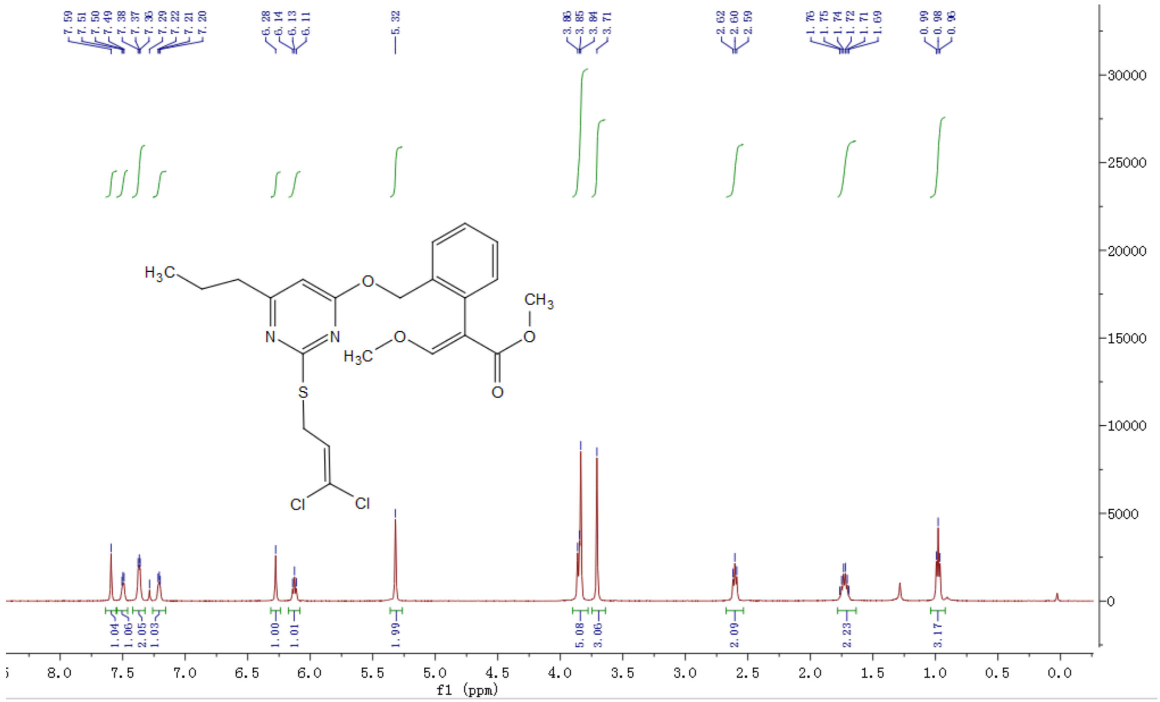

Fig. S33 ^{1}H NMR of compound **4t**

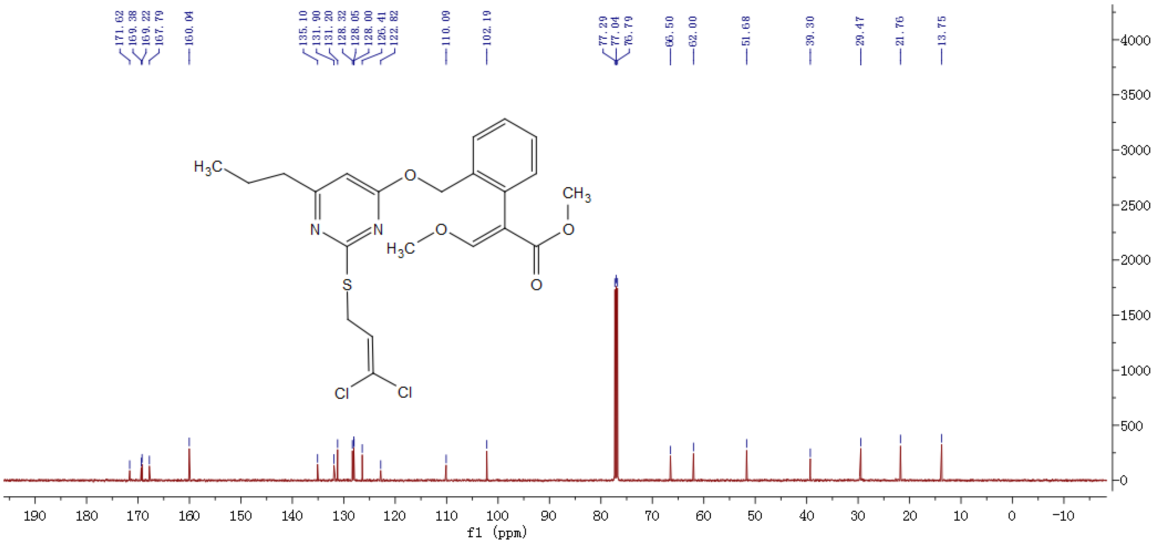

Fig. S34 ^{13}C NMR of compound **4t**

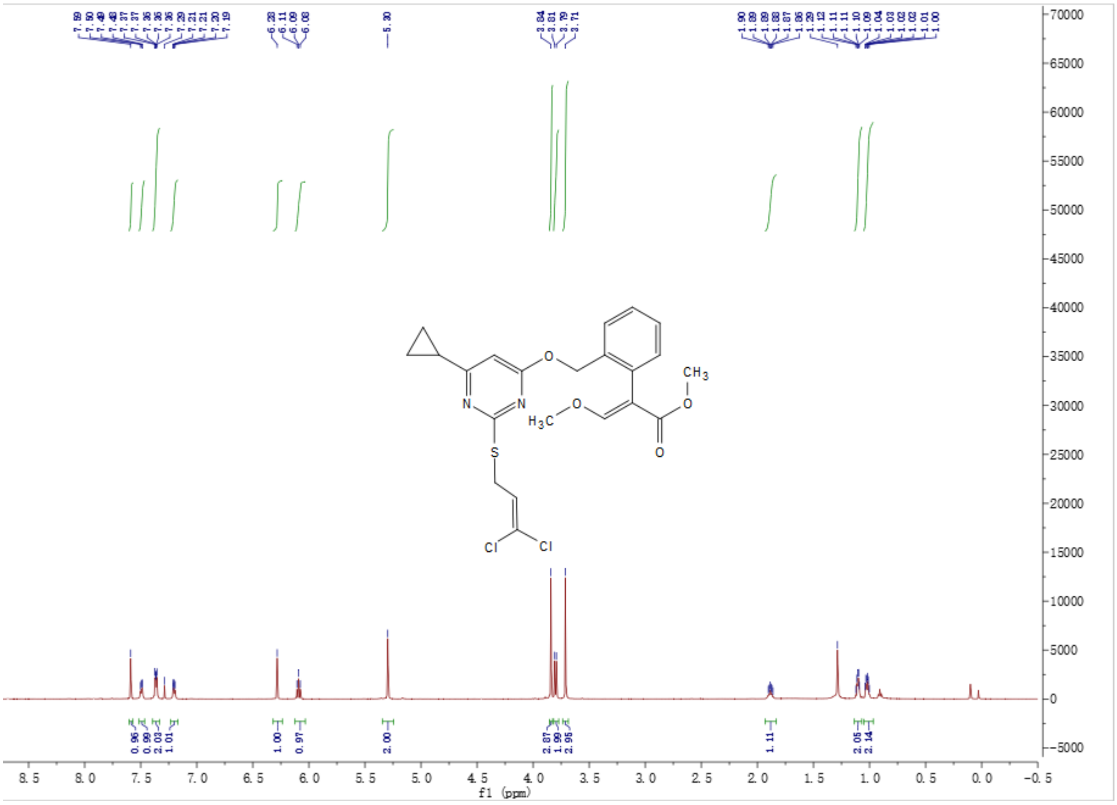

Fig. S35 ^{1}H NMR of compound **4u**

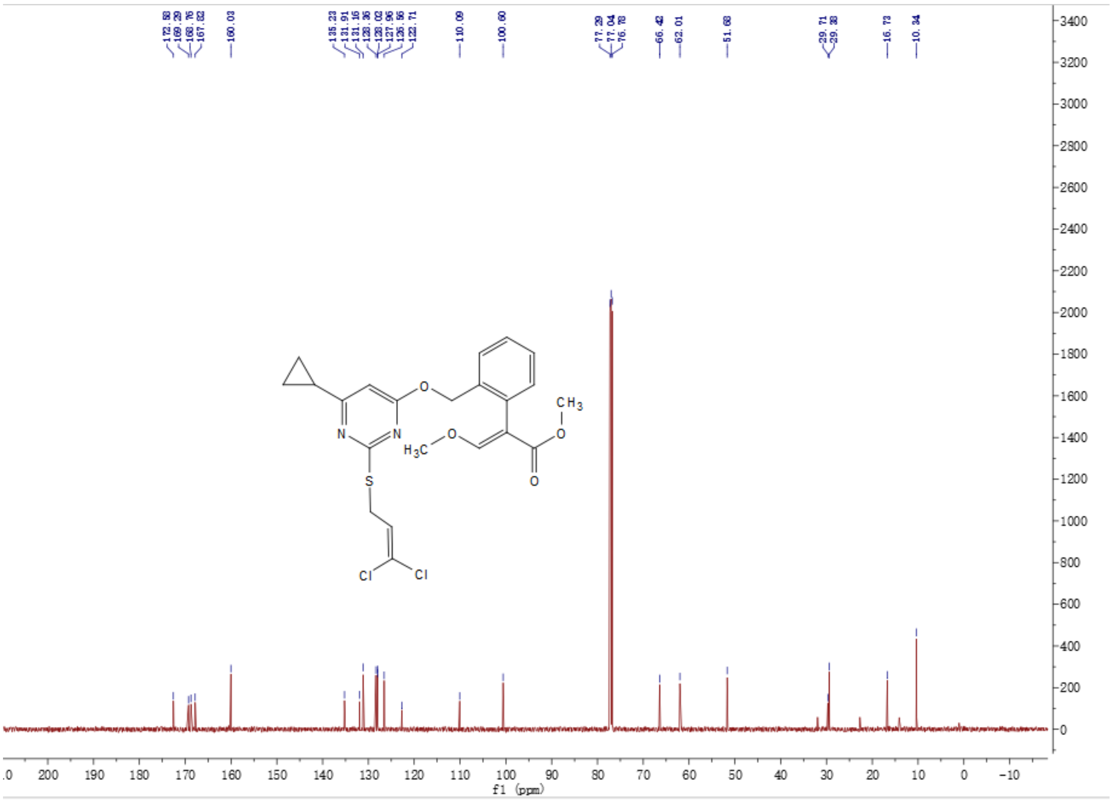

Fig. S36 ^{13}C NMR of compound **4u**
